# Quartz-Enhanced Photoacoustic Spectroscopy: A Review

**DOI:** 10.3390/s140406165

**Published:** 2014-03-28

**Authors:** Pietro Patimisco, Gaetano Scamarcio, Frank K. Tittel, Vincenzo Spagnolo

**Affiliations:** 1 Department of Electrical and Computer Engineering, Rice University, 6100 Main Street, Houston, TX 77005, USA; E-Mail: fkt@rice.edu; 2 Dipartimento Interateneo di Fisica, Università degli studi di Bari Aldo Moro e Politecnico di Bari, Via Amendola 173, Bari, I-70126, Italy; E-Mails: pietro.patimisco@uniba.it (P.P.); gaetano.scamarcio@uniba.it (G.S.)

**Keywords:** quartz enhanced photoacoustic spectroscopy, quartz tuning fork, gas sensing, mid-IR and THz laser spectroscopy

## Abstract

A detailed review on the development of quartz-enhanced photoacoustic sensors (QEPAS) for the sensitive and selective quantification of molecular trace gas species with resolved spectroscopic features is reported. The basis of the QEPAS technique, the technology available to support this field in terms of key components, such as light sources and quartz-tuning forks and the recent developments in detection methods and performance limitations will be discussed. Furthermore, different experimental QEPAS methods such as: on-beam and off-beam QEPAS, quartz-enhanced evanescent wave photoacoustic detection, modulation-cancellation approach and mid-IR single mode fiber-coupled sensor systems will be reviewed and analysed. A QEPAS sensor operating in the THz range, employing a custom-made quartz-tuning fork and a THz quantum cascade laser will be also described. Finally, we evaluated data reported during the past decade and draw relevant and useful conclusions from this analysis.

## 1. Introduction

The detection and measurement of trace gas concentrations is important for both the understanding and monitoring of a wide variety of applications, such as environmental monitoring, industrial process control analysis, combustion processes, detection of toxic and flammable gases, as well as explosives. For example, trace gas sensors capable of high sensitivity and selectivity are required in atmospheric science for the monitoring of different trace gas species including greenhouse gases and ozone, and in breath diagnostics, nitric oxide, ethane, ammonia and numerous other biomarkers. Quantitative and qualitative gas sensors can be categorized into four general groups: analytical sensors (principally gas-chromatography and spectrometry), electrochemical, semiconductor sensors and laser optical absorption sensors. The sensor classification is primarily based on the physical mechanism that is used. Analytical techniques do not offer real-time response, tend to be costly, invasive and occupying a large spatial footprint. Electrochemical gas sensors can be relatively specific to individual gas, have usable resolutions of less than one part per million (ppm) of gas concentration, and operate with very small amounts of current, making them well suited for portable, battery powered instruments [1]. However, they experience hysteresis and are influenced by water humidity. Moreover, one important characteristic of electrochemical sensors is their slow time response: when first powered up, the sensor may take several minutes to settle to its final output value and when exposed to a mid-scale step in gas concentration, the sensor may take tens of seconds to reach 90% of its final output value. Techniques based on laser absorption spectroscopy (LAS) for trace gas sensing, compared to other techniques, are considerably faster with response times of <1 s, suffer from minimal drift, offer high gas specificity, capable of part-per-quadrillion (ppq) detection sensitivity [2] and permit real time *in-situ* measurements. The principle of molecular absorption is based on the transitions that an electromagnetic wave causes in a chemical species. If a molecule is irradiated by infrared light, it is excited to a rotational-vibrational energy level manifold. Absorption lines are specific for each chemical species. To-date LAS has been developed mostly in the spectral region from 3 to 12 μm, which covers a substantial spectral range of fundamental transitions in the so called molecular finger-print region. Further extension into the vibrational overtone (1–2.5 μm), electronic (UV-Vis) and rotational (THz range) spectral range is also feasible.

The detection sensitivity of spectroscopic sensor systems that employ semiconductor lasers as a light source is limited by the available optical power, especially when dispersive elements and multi-detector arrays are used to analyze the light transmitted through the gas sample. In the near-IR spectral range optical parameter oscillators or conventional diode laser are typically the light source of choice. In the mid-IR and THz spectral regions quantum cascade lasers (QCLs) and interband cascade lasers (ICLs) are the optimal choice, due to their high output power, compactness, narrow spectral linewidth and broad wavelength tunability [3–5]. Two main QCL configurations are used: (1) distributed feedback (DFB) QCLs having a Bragg reflector built on top of waveguide, which forces the QCL to operate in single axial mode operation. QCLs are tuned by keeping the device operating temperature fixed and changing the current or vice versa, keeping the current fixed and changing the temperature; or (2) an external cavity QCL (EC-QCL), in which the quantum cascade device is the laser gain medium and mirrors are arranged in a configuration external to the laser to create an optical cavity. By replacing one of the external cavity mirror with a high quality diffraction grating, it is possible to tune the QCL emission wavelength over >15% of its central value.

LAS-based techniques offer not only excellent sensitivity and selectivity, but also long effective optical pathlengths, compactness, mechanical stability, versatility and cost effectiveness. In the case of cavity ring down spectroscopy (CRDS) an optical cavity with two concave mirrors with low loss and high reflectivity (>99.9%) provides a long optical path of up to several kilometers. A light pulse is injected into the cavity through one of the mirrors and inside the cavity, multiple reflections occur. After each reflection, leakage radiation from the cavity is registered by means of an appropriate photodetector [6]. A modification of the CRDS is cavity enhanced absorption spectroscopy (CEAS) in which the radiation is injected at a very small angle respect to the cavity axes which results in the formation of a dense structure of weak optical axial modes that makes the entire system more robust against instability in both the cavity and laser spectrum [7]. The idea of integrated cavity output spectroscopy (ICOS) is similar to CEAS. However, the measurement procedure is based on the comparison between the signal amplitude both at the input and the output of the cavity [8]. Both techniques require precise information about mirror reflectivity, a sensitive photodetector with a fast-response, perfect optical alignment and the use of long optical pathlengths. One of the most robust and sensitive trace-gas optical detection techniques is photo-acoustic spectroscopy (PAS), which is capable of extremely high detection sensitivities with a compact and relatively low-cost absorption detection module (ADM) [9].

## 2. Photoacoustic Spectroscopy

This technique is also based on an optical absorption process, such as CRDS, ICOS and CEAS, but differs in the physical phenomenon used for the detection of the absorption signal. When light at a specific wavelength is absorbed by the gas sample, the excited molecules will subsequently relax to the ground state either through emission of photons or by means of non-radiative processes. These processes produce localized heating in the gas, which in turn results in an increase of the local pressure. If the incident light intensity is modulated, the generation of thermal energy in the sample will also be periodic and a pressure wave, *i.e.*, a sound wave, will be produced having the same frequency of the light modulation. The PAS signal can be amplified by tuning the modulation frequency to one of the acoustic resonances of the gas sample cell. The key advantage of this technique is that no optical detector is required and the resulting sound waves can be detected by a commercial hearing aid microphone.

The photoacoustic signal *S* can be expressed by the relation:

(1) S=C⋅P⋅α

where *C* is the instrumental constant, *P* is the laser power and α is the absorption coefficient that is equal to:

(2) $$\alpha =N_{\text{tot}}\cdot \sigma \cdot c$$

where *σ* is the cross section of the optical transition, *c* is the concentration of the target gas and *N_tot_* is the total number of molecule per unit volume. From Equation (1) it follows that there is linear relationship between the sample concentration and the photoacoustic signal. The minimum optical absorption coefficient *α_min_* detectable with a PAS based sensor is determined by the condition *S* = *N*, where *N* is the noise level, which is assumed to be independent from the optical excitation. Hence, the minimum detectable concentration *c_min_* can be expressed by using Equation (2) as:

(3) $$c_{\text{min}}=\frac{\alpha_{\text{min}}}{N\cdot \sigma }$$

The instrumental constant *C* in Equation (1) depends on the cell size and geometry, the modulation frequency of the radiation, the efficiency of the transducer and the quality factor *Q* of the acoustic resonance defined by:

(4) $$Q=\frac{f_{0}}{\Delta f_{\text{FWHM}}}$$

where *f_0_* and Δ*f_FWHM_* are the resonant frequency and the full width at half maximum (*FWHM*) of the resonance profile, respectively. The quality factor *Q* can be experimentally measured and typically ranges from 40 to 200 and the resonant frequency from the measured values of Q and *f_0_* typically fall in the ranges 40–200 and 1,000–4,000 Hz, respectively. The PAS signal is proportional to the effective integration time *t* = *Q*/*f_0_*. One of the highest reported values is *t* = 56 ms. Achieving longer integration times in a gas-filled resonator is problematic because of intrinsic losses related to gas viscosity and other relaxation processes.

Continuous-wave single-mode diode lasers and optical parameter oscillators in the near-IR and QCLs in the mid-IR have been successfully applied in PAS [9]. Compact photoacoustic gas sensors based on broadband IR sources have been reported [10]. Resonant PAS cells and optical fiber amplifiers have been developed to enhance the PAS detection sensitivity [11]. The three main noise sources are: (i) noise caused by the radiation that is incident upon the walls of the PAS absorption cell; (ii) non-selective absorption of the gas cell window, and (iii) external acoustic noise. In order to improve the signal-to-noise ratio (SNR), different designs for PAS cells have been proposed and implemented including a resonant cell with acoustic buffers [12], windowless and a differential cell. A differential cell includes two acoustic resonators equipped with microphones having the same responsivity at the resonance frequency of the cell. Since the laser light excites only one of the two resonators, the difference between the two signals removes noise components that are coherent in both resonators [13].

PAS has been successfully applied in trace gas sensing applications, which include atmospheric chemistry, volcanic activity, agriculture, industrial processes, workplace surveillance, and medical diagnostics. For instance, PAS has been used to monitor nitric oxide (NO) from vehicle exhaust emissions, which contributes to respiratory allergic diseases, inflammatory lung diseases, bronchial asthma and the depletion of ozone [14]. In medicine, PAS has been used to monitor drug diffusion rates in skin [15] and to detect trace concentrations of disease biomarkers, such as ethylene (C_2_H_4_), ethane (C_2_H_6_), and pentane (C_5_H_12_), which are emitted by UV-exposed skin [16]. Other applications include monitoring respiratory NH_3_ emission from cockroaches as well as detecting the intake of prohibited substances by athletes [17]. Low cost portable PAS sensors have been on the market, examples of which include smoke detectors, toxic gas monitoring, and oil sensors for monitoring hydrocarbons in water.

## 3. Quartz-Enhanced Photoacoustic Spectroscopy

Quartz-enhanced photoacoustic spectroscopy (QEPAS) is an alternative approach to photoacoustic detection of trace gas utilizing a quartz tuning fork (QTF) as a sharply resonant acoustic transducer to detect weak photoacoustic excitation and allowing the use of extremely small volumes [18,19]. Such an approach removes restrictions imposed on the gas cell by the acoustic resonance conditions. A quartz crystal is a natural candidate for such an application because it is a low-loss and low-cost piezoelectric material. High-Q quartz crystals are employed as a frequency standard in clock, watches and smart phones. Usually QTFs with a resonant frequency of 2^15^ or ∼32,768 Hz are used. QTFs possess a Q ≈ 100,000 or higher when encapsulated in vacuum and a Q ≈ 10,000 at normal atmospheric pressure. Therefore, the corresponding energy accumulation time at atmospheric pressure is t ≈ 320 ms.

Acoustically, QTF is a quadrupole, which provide good environmental noise immunity. In fact, the width of the QTF resonance at normal pressure is ∼4 Hz, so only frequency components in this narrow spectral band can produce efficient excitation of the QTF vibrations. Sound waves in air at 32 kHz have an acoustic wavelength ∼1 cm, and thus, if produced by external acoustic sources, such waves tend to apply a force in the same direction on the two QTF prongs positioned at a ∼1 mm distance. As a result, such sound waves do not excite the piezoelectrically active mode in which the two prongs move in opposite direction and zero electrical response is produced. Hence, there is only one way to cause the QTF to resonate via the photoacoustic effect to produce sound waves from a source located between the two QTF prongs. The standard way to realize such a condition is for the excitation laser beam to pass through the gap between the prongs without touching them.

The generation of a photoacoustic wave involves the energy transfer from internal to translational molecular degrees of freedom. If a rotational-vibrational state is excited, a collision-induced vibrational to translation (V-T) relaxation follows, with a time constant that for a particular molecule is dependent on the presence of other molecules and intermolecular interactions. QEPAS measurements are usually performed at a detection frequency of about 32 kHz and are more sensitive to the V-T relaxation rate compared to the conventional PAS which is commonly performed at *f_0_* < *4* kHz. In case of slow V-T relaxation with respect to the modulation frequency, the thermal waves in the gas cannot follow fast changes of the laser induced molecular vibration excitation. Thus, the generated photoacoustic wave is weaker than it would be in case of fast V-T energy equilibration [20]. For instantaneous V-T relaxation, the detected photoacoustic signal can be expressed in the same way of the PAS:

(5) $$S\propto \frac{Q\cdot P\cdot \alpha }{f_{0}}$$

*Q* typically ranges from 10^4^ to 10^5^, depending on the carrier gas and the gas pressure.

### 3.1. QEPAS Sensor

**Figure 1.**
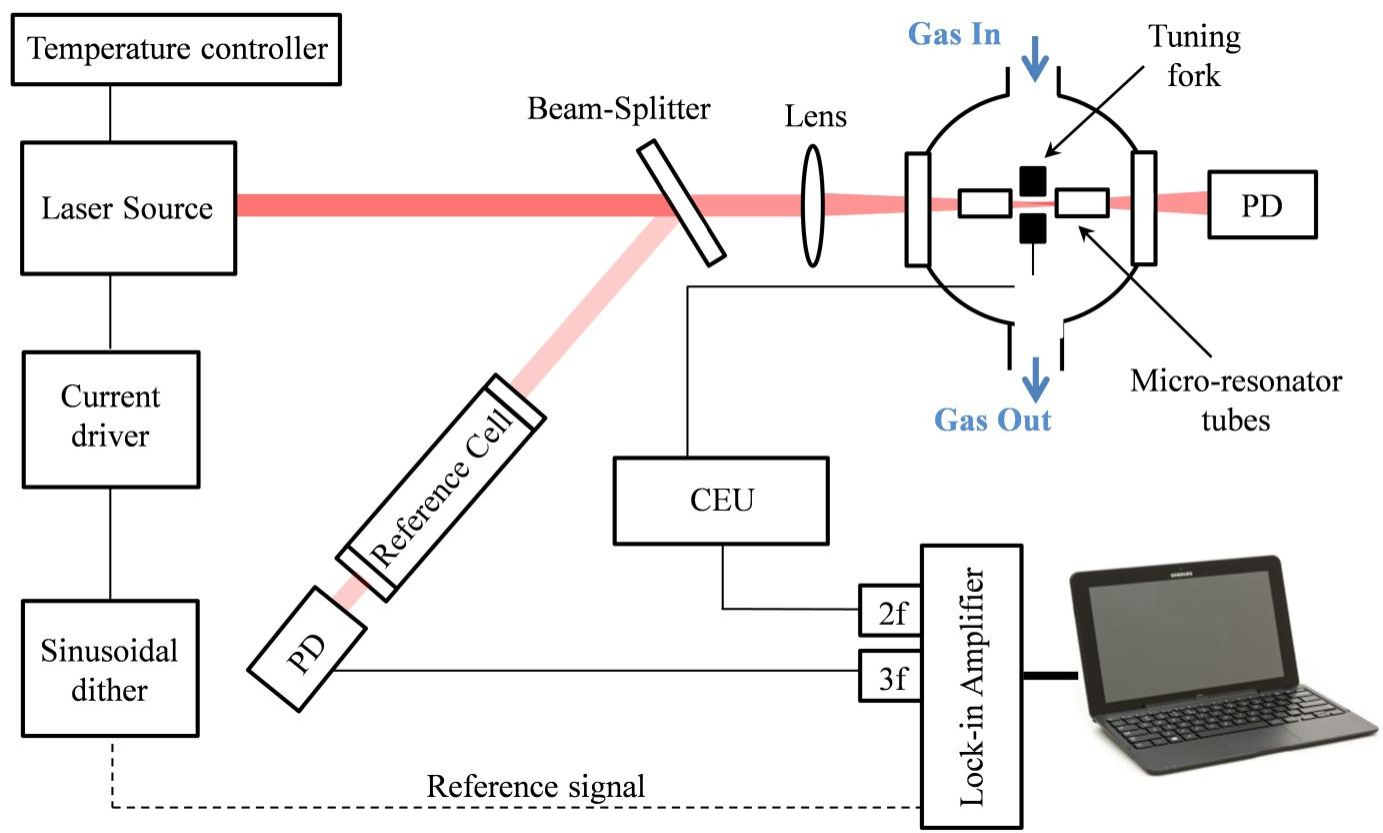
Schematics of a standard QEPAS based sensor. PD – Photodetector, CEU – Control Electronic Unit providing laser current and temperature, wavelength tuning & and two lock-in detection circuits.

A sketch of a typical QEPAS sensor, originally proposed in [18–20] and used in most reported QEPAS sensor systems, is shown in Figure 1. The optical components can vary depending on the spectral range, target molecule and the excitation laser. Typically, the wavelength of the laser is varied by changing the driving current when the temperature of the laser is fixed. This is the configuration used if employing for example a DFB QCL as the light source, with an integrated Peltier cooler and a temperature controller system. When an EC-QCL is used, both temperature and current are fixed, and the optical frequency can be scanned by applying a modulated voltage to a piezoelectric translator (not shown in the figure) attached to the diffraction grating element of the EC-QCL.

A beam-splitter can be used to split the incoming laser beam, sending a small portion to a reference cell full with a high concentration of the targeted trace gas. The light exiting the reference cell is detected by a photodetector and the absorption signal demodulated at *3f* by means of a lock-in amplifier. In this way, a *3f* wavelength locking technique can be implemented to lock the laser wavelength to the absorption peak line of the targeted molecule. The *3f* component crosses zero at the line center and is linear with detuning. If the detuning is sufficiently small, this feedback signal is used in a wavelength stabilization closed-loop. The laser beam passes through the beam-splitter and is focused between the two prongs of the QTF normally by using a lens. The wavelength modulation technique is implemented by applying a sinusoidal dither to the laser current at half of the QTF resonance frequency; a control electronic unit (CEU) is used to acquire the signal from the QTF that is demodulated at the QTF resonance frequency by means of a lock-in amplifier. The lock-in amplifier is usually controlled via a PC or a laptop computer through a USB NI card or RS232 communication.

#### 3.1.1. Quartz Tuning Fork: Resonant Properties and Noise

**Figure 2.**
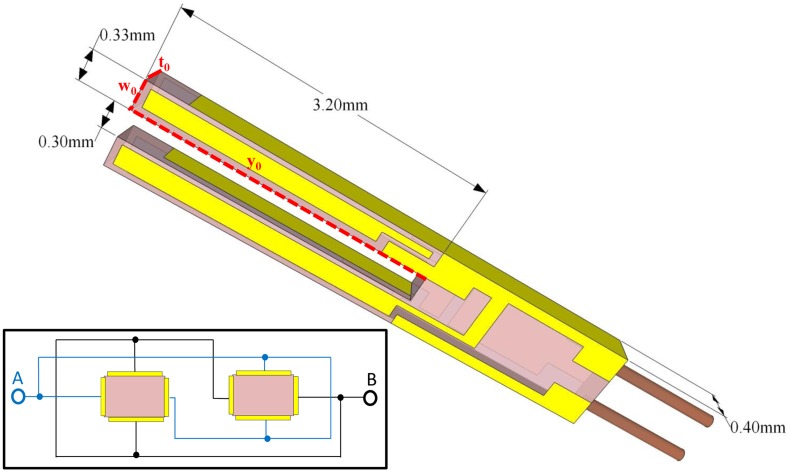
Schematic of a quartz tuning fork. Each prong can be modeled as a rectangular bar of dimension *w* × *y_0_* × *t*_0_ (dotted lines). Inset: a top view of the tuning fork with the electrical configuration for the electrodes A and B.

QTFs can be designed to resonate at any frequency in the 4–200 kHz range and beyond, since resonance frequencies are defined by the properties of the piezoelectric material and by its geometry. The interaction between the laser modulated beam and a trace gas leads to the generation of acoustic waves that mechanically bend the QTF prongs. Hence, the electrode pairs of the QTF will be electrically charged due to the quartz piezoelectricity. Piezoelectricity is the coupling between internal dielectric polarization and strain, and is present in most crystals lacking a center of inversion symmetry. When a stress is applied to these materials, it induces a displacement of charge and a net electric field. The effect is reversible: when a voltage in applied across a piezoelectric material, it is accompanied by a strain. Due to this intrinsic coupling of strain and charge displacement a QTF can be modeled both electrically and mechanically, each prong being modeled as a slab of dimension *w* × *y_0_* × *t_0_*, as shown in Figure 2.

**Figure 3.**
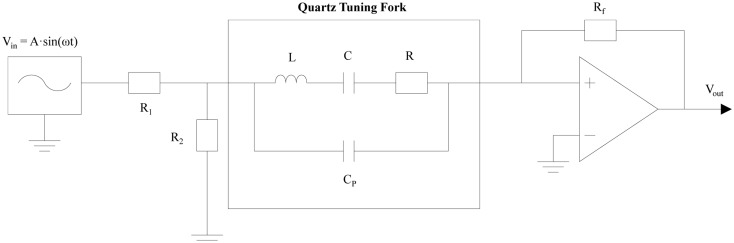
Schematic of the circuit used to characterize a QTF.

Both mechanical motion and electrical response can be modeled using differential equations, having equivalent mathematical forms. Thus, the QTF is both a circuit with capacitance *C*, resistance *R* and inductance *L*, and equivalently a mass *m* on a spring, with spring constant *k* and damping factor *β*. The two domains can be coupled through a relation, in which the force driving the QTF is proportional to the driving voltage. Hence, a voltage signal measured from the QTF can easily translate into the force on it. The circuit schematic used to characterize the QTF is shown in Figure 3. An optimum approach is to acquire the QTF electrical response using an ultra-low transimpedance amplifier with feedback resistor R_f_ = 10 MΩ. Feedback maintains a zero voltage between the QTF electrodes and so that the influence of the parallel stray capacitance *C_p_* is neutralized.

In this condition, the QTF model is reduced to an *RLC* series circuit and the resonant frequency is given by:

(6) $$f_{0}=\frac{1}{2\pi }\sqrt{\frac{1}{LC}}$$

and the *Q* factor is:

(7) $$Q=\frac{1}{R}\sqrt{\frac{L}{C}}$$

while the impedance of the *RLC* circuit at the resonance condition is equal to its resistance.

The QEPAS sensor noise measured at the amplifier output at the resonant frequency *f_0_* is primarily determined by the thermal noise of the equivalent resistor *R* [17]:

(8) $$\frac{\sqrt{\langle {V_{N}}^{2}\rangle }}{\sqrt{\Delta f}}=R_{f}\sqrt{\frac{4KT}{R}}$$

where *V_N_* is the voltage noise at the transimpedance amplifier output, *Δf* is the detection bandwidth and *T* is the QTF temperature. The feedback resistor also introduces noise, which is several times lower than the thermal QTF noise and can be neglected for typical values of *R* in the range 10–100 KΩ. The electrical parameters of the QTF can be determined by using a CEU, which applies an ac voltage *V* to the circuit depicted in Figure 3, while scanning the applied voltage frequency *f*. The maximum of the *I(f)* function, where *I* is the QTF current and *f* is the modulation frequency of the applied voltage, yields the resonant frequency *f_0_*. In this way, R and Q are determined by using the Equation (7) and the relation:

(9) $$R=\frac{V}{I(f_{0})}$$

**Figure 4.**
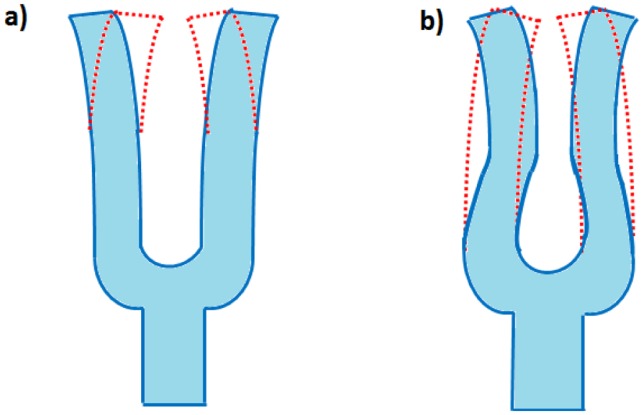
(**a**) First in-plane vibrational mode of a tuning fork. (**b**) Third in-plane vibrational mode of a tuning fork.

The available resonant frequencies of the QTF can be calculated by considering an approximation in which each prong of the fork is considered to behave as a cantilever vibrating in the in-plane flexural modes of the tuning fork. The first two flexural modes are shown in Figure 4.

In the first flexural mode, the tines move in opposite directions and the center of mass of the QTF remains unchanged. The flexural mode vibration can be modeled by considering that each prong of the tuning fork behaves as a clamped beam. When the force is removed from the displaced beam, the beam will return to its original shape. Assuming the elastic modulus, inertia and cross sectional area are constant along the beam length, the equation for that vibration is given by the following fourth-order differential equation, according to the Euler-Bernoulli approximation:

(10) $$EI\frac{\partial^{4}y}{\partial x^{4}}\left(x,t\right)+\rho A\frac{\partial^{4}y}{\partial t^{4}}\left(x,t\right)=0$$

where *ρ* is the density of the material, *E* the Young modulus of the material, *t* is the time, *A* = *w* × *y_0_* and *x* and *y* directions in the plane of the QTF. Equation (10) can be solved by separation of variables, assuming that the displacement can be separated into two parts; one that depends on position and the other on time. This leads to a simplified differential equation for the *y* direction that can be solved by superimposing boundary conditions. These boundary conditions come from the support of the QTF. The fixed end must have zero displacement and zero slope due to the clamp, while the free end cannot have a bending moment or a shearing force (free-clamped boundary conditions). The general solution is a linear combination of trigonometric equations leading to the frequency equation for the cantilever beam [21]:

(11) $$\cos\left(k_{n}y_{0}\right)\cosh\left(k_{n}y_{0}\right)=-1$$

where *k_n_* are the wavenumbers related to the eigenfrequencies *f_n_*, given by the following expression:

(12) $$f_{n}=\frac{\pi K}{8{y_{0}}^{2}}\sqrt{\frac{E}{\rho }}n^{2}$$

where $K=(\frac{1}{\sqrt{12}})w_{0}$ and $n={\left(\frac{\rho A}{EI}{\omega_{n}}^{2}\right)}^{\frac{1}{4}}$. U-shaped QTFs are mass-produced as mentioned previously on page 5 for timing application used in electronic clocks and smartphones. The standard QTF has a resonant frequency of ∼32 KHz, with a gap between the prongs of ∼300 μm, and prongs that are 3.2 mm long and 0.33 mm wide, as depicted in Figure 2. Such QTFs have been widely used in all mid-IR QEPAS based sensors reported in the literature to-date, because of their commercial availability and ultra-low cost. Chromium/gold layer are deposited on both sides of the QTF to create electrodes, which collect the electrical charges induced by the mechanical deformation. The first three solutions of Equations (11) and (12) are shown in Table 1:

When a QTF vibrating at harmonics oscillations of small amplitude is immersed in a fluid medium, it tends to induce a motion in the fluid, which gives rise to an energy loss and additional inertia. The reaction force is hence composed of a resistive part, which leads to energy dissipation by acoustic loss and a reactive part, which gives rise to an additional inertia to the vibrating QTF body. For these conditions, Equation (10) can be modified by considering a term which includes damping effects (damping parameter *C_d_*) and an added mass *u* per unit length [21]:

(13) $$EI\frac{\partial^{4}y}{\partial x^{4}}\left(x,t\right)+C_{d}\frac{\partial y}{\partial t}\left(x,t\right)+(\rho A+u)\frac{\partial^{4}y}{\partial t^{4}}\left(x,t\right)=0$$

By assuming in a first approximation that damping remains small and *u* ≪ *ρA* it can be shown that an added mass causes a shift of the QTF resonance frequency, Δf with respect to that in vacuum:

(14) $$\Delta f=-\frac{1}{2}\frac{u}{\rho A}$$

The exact derivation of the added mass is a complicated problem even for a simple structure. For a vibrating prong in steady motion, the added mass is proportional to the density *ρ_0_* of the fluid medium. Since there is a linear relationship between the pressure and the density in a gas, we obtain that *Δf* varies linearly with gas pressure. However, the fluid damping influences negatively the quality factor *Q* of the flexural beam. In fact, the reaction force due to the presence of the gas acting on a vibrating QTF leads to energy dissipation by means of an acoustic loss. Assuming that the fluid drag force is the dominant damping source, *i.e.* the resonator is isolated from other objects so that other damping effects need not to be considered, and the fluid damping parameter can be expressed as [22]:

(15) $$C_{d}\propto \sqrt{P\mu_{0}}$$

where *μ_0_* is the viscosity of the fluid and where the linearity between the density and the pressure in a gas applies again. Hence the influence of fluid damping on the *Q* can be expressed in terms of the energy loss *1*/*Q(P)* at a gas pressure, *P*:

(16) $$C_{d}=\frac{1}{Q(P)}-\frac{1}{Q_{0}}$$

where *Q_0_* is the tuning fork *Q* factor in vacuum, which depends only on the internal losses and losses added by the electrical circuit of the transimpedance amplifier. By using Equation (15), *Q(P)* can be described as:

(17) $$Q\left(P\right)=\frac{Q_{0}}{1+Q_{0}a\sqrt{P}}$$

Hence, the dependence of QEPAS detection sensitivity upon the trace gas concentration in a specific absorbing gas sample is a function of the sample pressure. This dependence is influenced by different parameters: (i) the *Q* factor of a QTF decreases rapidly with increasing pressure due to energy losses by the mechanical viscosity and friction losses via the trace gas; (ii) at low gas pressures the collisional line broadening of the absorption peak is less than Doppler broadening Hence, the merging of closely spaced absorption lines should be taken into account at higher pressures; (iii) V-T relaxation rates are faster at higher pressures which is in competition with the opposite trend of the *Q* factor; and (iv) the speed of sound depends on the gas pressure.

#### 3.1.2. Wavelength Modulation Detection

The wavelength modulation (WM) technique is generally used to improve the QEPAS SNR and to minimize external acoustic noise for a QEPAS based sensor system. The WM description is based on an intensity representation of an optical wave, so that only the absorption of the sample is considered and dispersion effects due to the sample can be neglected. The description is based on the instantaneous laser frequency:

(18) $$\upsilon \left(t\right)=\nu_{0}-\Delta \nu \text{cos}(\omega t)$$

where υ_0_ is the optical carrier frequency and *ω* = *2πf* is the modulation angular frequency due to the laser injection current modulated at the same angular frequency. In addition to frequency modulation, the current waveform applied to the QCL produces a sinusoidal modulation of the laser intensity and is given by:

(19) $$I\left(t\right)=I_{0}+\Delta \text{Icos}(\omega t)$$

The amplitude *ΔI* of the sinusoidal intensity modulation is determined by the slope of the laser power *versus* the current characteristics, which is assumed constant across a wavelength scan. The instantaneous laser frequency interacts with the absorption feature. Expanding the absorption coefficient *α(ν(t))* for a small *Δν* we obtain:

(20) $$\alpha \left(\upsilon \left(t\right)\right)=\alpha_{0}+{\frac{\partial \alpha }{\partial v}|}_{\upsilon =\nu_{0}}\Delta \nu \cos\left(\omega t\right)+\frac{1}{2}{\frac{\partial^{2}\alpha }{\partial v^{2}}|}_{\upsilon =\nu_{0}}{\left(\Delta \nu \right)}^{2}\cos^{2}\left(\omega t\right)+...$$

where *α_0_* can be considered to be the background absorption contribution. The laser is modulated both in intensity and in wavelength simultaneously. Thus, assuming a small absorption *I_abs_*, from the Lambert-Beer law we have:

(21) $$I_{\text{abs}}\left(t\right)=\left[I_{0}+\Delta \text{Icos}\left(\omega t\right)\right]\left[1-L\left(\alpha_{0}+{\frac{\partial \alpha }{\partial v}|}_{\upsilon =\nu_{0}}\Delta \nu \cos\left(\omega t\right)+\frac{1}{2}{\frac{\partial^{2}\alpha }{\partial v^{2}}|}_{\upsilon =\nu_{0}}{\left(\Delta \nu \right)}^{2}\cos^{2}\left(\omega t\right)\right)\right]$$

*L* is the effective length over which the absorption takes place to produce an acoustic wave detectable by the QTF (in other words, *L* is comparable with the thickness *t_0_* of the QTF. Hence, the *1ω*-signal, *S_1ω_*, is given by:

(22) $$S_{1\omega }=L\Delta I\alpha_{0}-L{\frac{\partial \alpha }{\partial v}|}_{\upsilon =\nu_{0}}\Delta \nu$$

and the *2ω*-signal *S_2ω_* is given by:

(23) $$S_{2\omega }=-L\Delta I{\frac{\partial \alpha }{\partial v}|}_{\upsilon =\nu_{0}}\Delta \nu +\frac{I_{0}}{2}{\frac{\partial^{2}\alpha }{\partial v^{2}}|}_{\upsilon =\nu_{0}}{\left(\Delta \nu \right)}^{2}$$

This result shows that the background absorption contributes to the *S_1ω_*, whereas it does not contribute to *S_2ω_*. If we assume that the absorption coefficient has a pure Lorentzian lineshape, *S_1ω_* has a pure first derivative line-shape with a constant background; *S_2ω_* consists of two terms: the first term, arising from a residual amplitude modulation is proportional to the first derivative, whereas the second is the second-derivative expression arising from the laser wavelength modulation. Hence, *S_2ω_* is not a pure second derivative of the Lorentzian lineshape but is distorted by a contribution originating from the residual amplitude modulation. This distortion does not affect the peak position of *S_2ω_* since the first derivative of the Lorentzian lineshape vanishes when *υ* = *υ_0_*.

**Figure 5.**
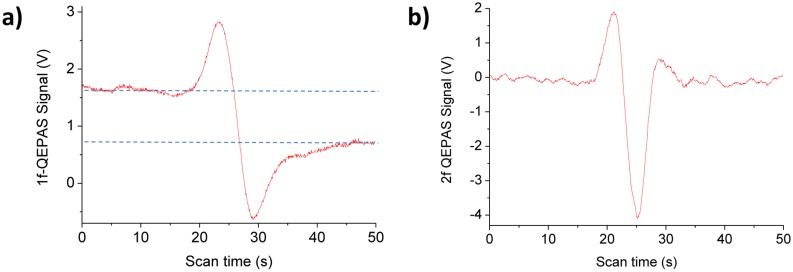
*1f-QEPAS* (a) and *2f-QEPAS* (b) spectral scans of 2.6 ‰ of CO_2_ in N_2_ at a gas pressure of 50 Torr of a CO_2_ line centered at 2,311.515 cm^−1^. Blue lines highlight the strong background of the spectral *1f-QEPAS* acquisition.

The generated QEPAS signal is usually demodulated by means of a lock-in amplifier both at the fundamental frequency *f* or the successive harmonics *nf*. When the laser light is modulated at the resonant frequency *f_0_* and QEPAS signal is demodulated at the same frequency, the demodulated signal is usually called *1f-QEPAS* signal, while when the laser light is modulated at the *f_0_/2* and QEPAS signal is demodulated at *f_0_*, the demodulated signal is referred to *2f-QEPAS* signal. In this case, the QTF detects sound oscillations at the second harmonic of the modulation frequency caused by the double intersection of the absorption line by the laser line during a modulation period. In Figure 5 *1f-QEPAS* (*2f-QEPAS*) spectral scan of a 2.6 ‰ of CO_2_ in N_2_ mixture is depicted at a total gas pressure of 50 Torr, obtained by current modulation at *f*_0_=32,809.1 Hz (*f*_0_/2=16,504.55 Hz) and a peak-to-peak voltage amplitude of 2.8 V acquired by using a bare QTF (*i.e.*, without mR tubes) and a CW DFB QCL targeting the CO_2_ (01^1^1)–(01^1^0) P(29) rotational-vibrational transition centered at 2,311.515 cm^−1^ and with a linestrength of 7.458 × 10^−20^ cm/mol.

A strong background signal was observed for the *1f* approach, originating from stray light ending up on the walls of the acoustic detection module. This is confirmed by the observation that the amplitude of this offset strongly increases with a misalignment of the laser beam in lateral directions so that the beam wings touch the QTF. Instead, it was experimentally observed that the *2f* approach is background-free. Distortions in the demodulated signal displaying an asymmetry on both side of the spectrum around the peak (see Figure 5b) can be associated to an amplitude intrinsic modulation contribution, which is introduced by current modulation. The laser wavelength modulation amplitude *Δf* and light intensity modulation *ΔI* must be optimized at each gas pressure for a highest *2f-QEPAS* signal.

#### 3.1.3. Amplitude Modulation Detection for Broadband Absorbers

Vibrational spectra of most molecules consisting of more than five atoms are so dense that infrared absorption spectra of such polyatomic molecules consist of 100–200 cm^−1^ broad bands. Spectroscopic identification of these species requires laser excitation sources with a wide spectral coverage. However, distributed feedback (DFB) or Fabry-Perot (FP) QCLs cannot be wavelength modulated with a sufficient spectral tuning coverage for broadband absorption features. Thus, QEPAS detection of such molecules will require amplitude modulation (AM) of the laser radiation. The laser is operated at *f_0_* by means of square wave amplitude current modulation and the QEPAS signals are detected by a lock-in amplifier at the same *f_0_* frequency. Unlike WM QEPAS, AM QEPAS is not background free. Residual absorption of laser radiation by the cell windows as well as scattered radiation absorbed inside the gas cell produce a sound at the TF resonant frequency, thus generating a coherent background. However, this background can be stable over several hours, which allows background subtraction. Typically, for every spectral point, both signal and background components normalized to the laser power are acquired. In post-processing, the in-phase and the quadrature components of the photoacoustic signal were calculated, respectively and by vector subtraction, it is possible to remove the background signal [22–25].

### 3.2. On-Beam QEPAS

**Figure 6.**
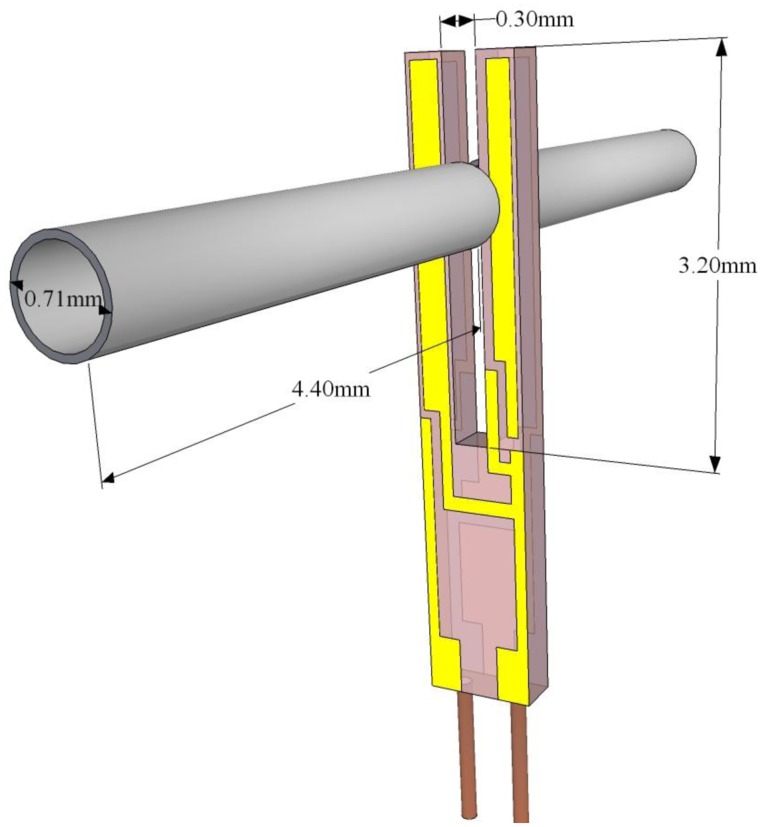
Schematic of an on-beam QEPAS configuration. Two micro-resonator tubes are aligned perpendicular to the QTF plane to probe the acoustic vibration excited in the gas contained inside the ADM.

To enhance the QEPAS signal and to confine the sound wave a micro-resonator (mR) is employed. The typical acoustic mR consists of two thin tubes aligned perpendicular to the QTF plane. The on-beam coupled resonator system as depicted in Figure 6 consisting of a QTF and a mR is called a spectrophone or acoustic detection module (ADM). This innovative approach is described in detail in [18–20,23–50]. It is critical that the laser beam that enters the mR does not touch the mR walls in order to avoid photothermal effects.

The QTF resonant parameters change when it is coupled to the mR. The decrease of the *Q* factor provides a measure of acoustic coupling between the QTF and the mR, since the high-*Q* QTF loses energy primarily via coupling to the low-*Q* mR oscillator. This can be explained by considering that a QTF is acoustically a quadrupole and the mR is an acoustic dipole with a higher acceptance bandwidth, which results in a reduction of the *Q* factor of the acoustically coupled system. A QTF integrated into a mR has a higher fundamental frequency indicating that an additional force constant is added due to acoustic coupling. If we consider in a first approximation that the two parts of the mR can be considered as a single tube, each tube cut to a length *l∼λ_s_/4*, where λ_s_ is the sound wavelength, behaves as a half-wave resonator. Further analysis and experimental studies have shown that the QEPAS signal is in fact stronger when *l∼λ_s_/2* suggesting that the gap between the tubes is big enough to make them quasi-independent. More detailed studies showed that the optimum length is in fact between *λ_s_/4* and *λ_s_/2* because of the interaction of mR tubes and acoustic coupling to the QTF [51,52].

Dong *et al.* [40] reported a detailed performance evaluation of the QEPAS sensor operation as a function of the geometrical mR parameters (tube diameter, tubes length and gaps between the QTF surface and tube end facets) and derive conclusions regarding the optimum mR configuration depending on the specific application, the operating pressure and the acoustic coupling. By a selective choice of the size, the acoustic resonance in the mR tubes can significantly improve the sensor performance. The best SNR of the QEPAS signal is achieved with tubes having a length of 4.4 mm, an internal diameter of 0.6 mm and an external diameter of 0.71 mm. The gaps between each side of mR tubes and the QTF surface were in the 30–50 μm range. This mR tubes length is significantly shorter than the half-wavelength of sound estimated by considering the speed of sound (340 m/s) in pure nitrogen at 293 K [38]. At the optimum tube length, the effects of acoustic resonance dominate and the *Q* factor of the mR filled with gas increases with its diameter. By using longer mR tubes, the SNR increases when the tube diameter is reduced and resonant effects in the mR are not strongly pronounced. Therefore, the volume of the excited gas becomes the primary factor and causes the QEPAS signal to increase when the absorbed optical energy is distributed over a smaller amount of gas. For a mismatched condition, the mR tubes simply confine the sound wave and do not exhibit a pronounced resonant behavior. By increasing the external diameter (thicker mR tubes) the acoustic coupling is higher, which result in a lower *Q* factor. Tubes with a larger inner diameter match the QTF frequency when reduced to a shorter length. In fact, with a larger tube diameter the gap between two mR tubes becomes less important and the system moves closer to the configuration in which we can neglect the gap between the two parts of the mR tubes (*l∼λ_s_/*4*)* [53]. This configuration has the advantage of easier optical alignment, but the spectrophone is more sensitive to environmental acoustic noise due to a lower *Q* factor. A record SNR improvement in SNR by a factor of ∼30 was achieved by using tubes having a length of 4.4 mm and an internal diameter of 0.6 mm, for a spectrophone operating in the pressure range 500–700 Torr [40].

**Figure 7.**
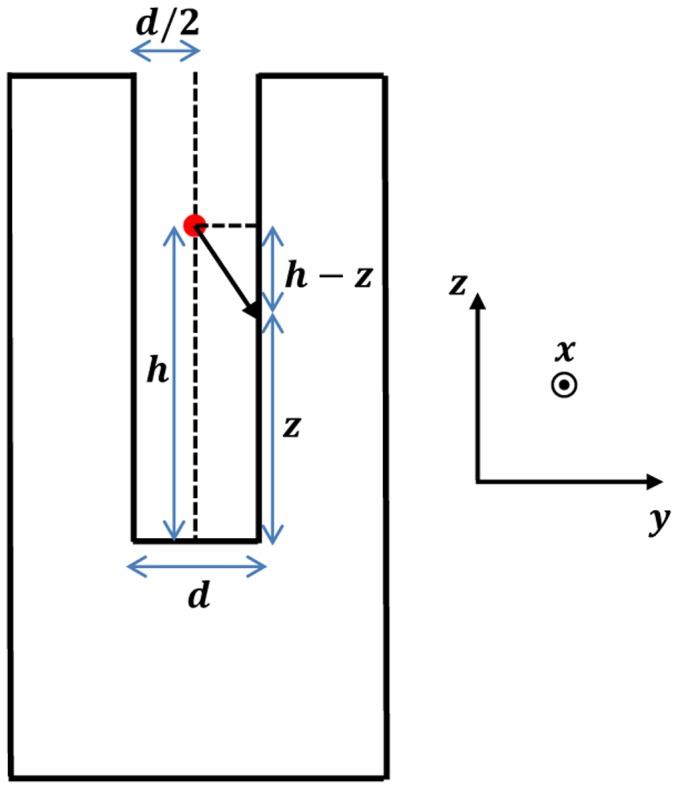
QTF with the main parameters employed for calculations and related to the coordinate system used. The origin of the y-axis is centered between the prongs.

In the following, a simplified model which considers the total momentum of a pressure force acting on the two prongs of the QTF is presented. The main assumptions are: (i) two prongs, which radiate as point sources and create divergent spherical-shaped sound waves (monopole approximation); (ii) the intensity of the pressure wave decreases as the inverse of the distance and is assumed to be constant along the thickness of each prong. In this approximation only force lines that lie on y-z plane (the plane that contains the two prongs) are considered; (iii) acoustic coupling between two oscillating prongs can be neglected (small oscillations approximation). The acoustic source is centered on the z-axis, as shown in Figure 7. By assuming that the beam waist of the focused laser beam is equidistant from the two prongs, the total momentum *M_tot_* can be expressed by the following relation:

(24) $$M_{\text{tot}}=2\int_{0}^{L}\frac{P_{0}t_{0}dz}{\sqrt{{\left(\frac{d}{2}\right)}^{2}+{\left(h-z\right)}^{2}}}\frac{\frac{d}{2}}{\sqrt{{\left(\frac{d}{2}\right)}^{2}+{\left(h-z\right)}^{2}}}z$$

where $\frac{P_{0}t_{0}dz}{\sqrt{{\left(\frac{d}{2}\right)}^{2}+{\left(h-z\right)}^{2}}}$ is the acoustic pressure on the infinitesimal area element *t*_0_ *dz* of the surface and $r=\sqrt{{\left(\frac{d}{2}\right)}^{2}+{\left(h-z\right)}^{2}}$ is the prong distance from the sound wave point source; $\frac{\frac{d}{2}}{\sqrt{{\left(\frac{d}{2}\right)}^{2}+{\left(h-z\right)}^{2}}}$ is the sine of the angle between the force vector acting on the prong and the lever arm; *P_0_* is the initial value of the intensity of the pressure wave; *d* is the gap between the two QTF prongs.

Upon integration, an expression for *M_tot_* as a function of *h* (the vertical position) can be found:

(25) $$M_{\text{tot}}=\text{cost}\left\{\frac{1}{4}ln\left[\frac{d^{2}+4{\left(h-y_{0}\right)}^{2}}{d^{2}+4h^{2}}\right]+\frac{h\left[tan^{-1}\left(\frac{2h}{d}\right)-tan^{-1}\left(\frac{2\left(h-y_{0}\right)}{d}\right)\right]}{d}\right\}$$

**Figure 8.**
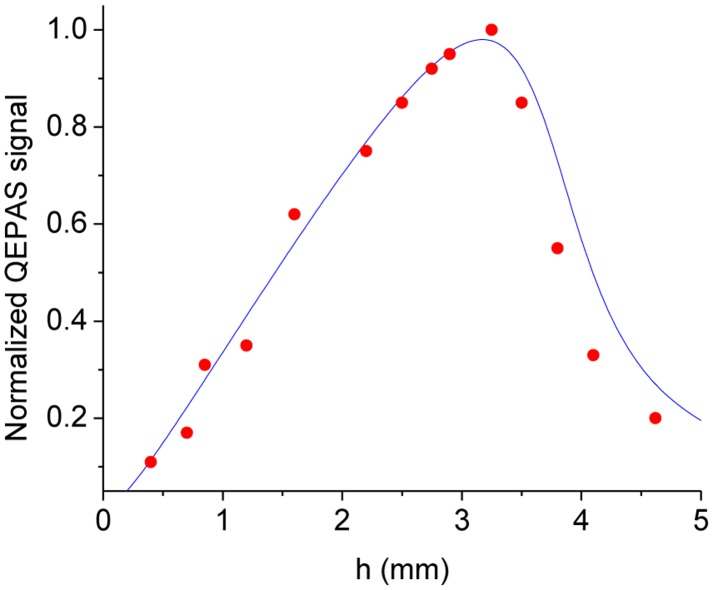
Normalized experimental results (dots) of the QEPAS signals as a function of the vertical position of the QTF. Data are obtained by detecting a concentration of 2% H_2_O in N_2_ at 75 Torr and by using an on-beam QEPAS sensor system configuration. The solid line is the best fit curve obtained using Equation (3.21).

To validate the model and study the dependence of the QEPAS signal strength (which is proportional to the total momentum generated by the pressure wave) as a function of the vertical position of the laser beam, the experimental data obtained by employing a QEPAS sensor with a 32.8 kHz QTF, was fitted using Equation (25). The experimental results were acquired by detecting H_2_O in N_2_ at a concentration of 2% of at a total gas pressure of 75 Torr using a peak-to-peak voltage amplitude of 4.2 V and a EC-QCL emitting at 10.54 μm for targeting a H_2_O line centered at 948.263 cm^−1^. The experimental data and the obtained best fit by using Equation (25) are shown in Figure 8.

The experimental data depicted in Figure 10 was normalized to the highest QEPAS signal. The theoretical model can thus accurately predict the experimentally observed optimal vertical position of the laser beam.

A more detailed and exhaustive theoretical model for the determination of the beam position of the laser beam that maximizes the QEPAS signal was proposed by Petra *et al.* [54]. The model consists of three stages. First, Petra *et al.* calculate an explicit formula for the acoustic pressure wave by using the cylindrical symmetry of the laser beam and the narrow width of the tuning fork resonance, to reduce the inhomogeneous wave equation to a Bessel equation. The model shows that the amplitude of the pressure wave is proportional to the laser modulation frequency. Then, they use the Euler-Bernoulli equation to model the resonant vibration of the prongs of the QTF. Finally, they use the well-known electromechanical relationships of for QTFs to calculate the piezoelectric current generated by the mechanical vibration. To derive analytical solution for this model, they ignored the effect the QTF has on the acoustic pressure wave and assumed that the piezoelectric response of the tuning fork can be obtained by modeling each prong individually. In spite of these simplifying assumptions, an excellent agreement between theory and experiments was found and the optimal vertical position of the focused laser beam occurs at *y ∼* 3.3 mm in good agreement with the result of our simplified model.

### 3.3. Off-Beam QEPAS

**Figure 9.**
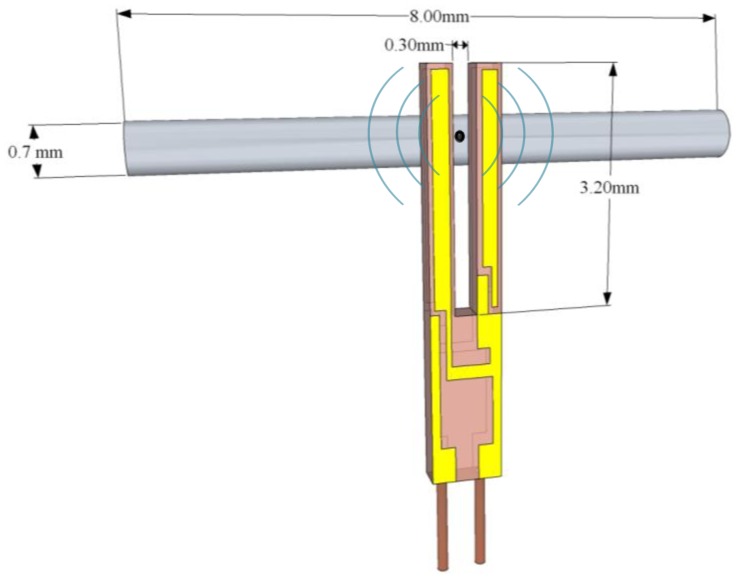
Sketch of an off-beam QEPAS spectrophone configuration. The micro-resonator tube is located close to the prongs of the QTF without touching them. The green circular lines indicate the acoustic wavefronts.

The on-axis QEPAS configuration has several disadvantages, such as: (i) the resonant acoustic wave condition was not exactly obtained; (ii) the open-ended tubes introduce sound energy-losses; (iii) the gap between the QTF prongs is only ∼300 μm wide, which limits the inner diameter of the mR and thus the size of the laser beam that passes through the tubes; An alternative configuration called “off-beam QEPAS” was first reported in [55]. In off-beam-QEPAS, the laser beam and the QTF are separated by a physical barrier, *i.e.*, the microresonator walls and the QTF senses the pressure in the microresonator through a small aperture in its center. Thus, in off-beam-QEPAS configuration, the mR is a single tube and its length determines the first longitudinal mode of the acoustic wave at *f_0_*. For this mode, the resonant acoustic pressure antinode is at the center of the mR. Hence a small slit is made in the middle of the mR and the QTF is coupled to the mR by placing it external to the mR tube close to the centrally located mR aperture. A sketch of the off-beam configuration is shown in Figure 9.

The off-beam-QEPAS configuration results in certain technical advantages as it facilitates the optical alignment and allows more flexibility in the selection of QTF dimensions. In off-beam-QEPAS, the acoustic oscillations of the gas are excited in the mR by the intensity modulation induced by the externally located laser source. The photoacoustic signal amplitude, *A* in the mR can be expressed as [56]:

(26) A=C(f)αP

where *C(f)* is a geometrical parameter which describes the characteristics of the mR at a given frequency *f*, that is usually the same as the QTF resonant frequency *f_0_* or its higher harmonics. The acoustic oscillations in the mR give rise to sound waves radiated via a slit at its center and are detected by the QTF placed outside the mR, close to the slit, The off-beam-QEPAS signal is generated at the QTF resonant frequency *f_0_*, which is proportional to the amplitude of the photoacoustic signal as described by Equation (3.22). To maximize sound energy coupling, the distance between the mR and the QTF must be carefully chosen, since too long a distance will decrease the acoustic coupling between mR and QTF, while too a short distance will dampen the QTF vibration because of viscous drag. K. Liu *et al.* [55] have shown that the close proximity of the QTF to mR results in optimum acoustic coupling, and therefore a higher QEPAS signal and SNR. At the same time, viscous drag in the air layer between the QTF and mR reduces the *Q* factor and the SNR increases up to a gap of 5 μm and then decreases for a wider QTF gap. The decrease of the QTF *Q* factor in the distance between mR and the QTF can be approximated by an exponential decay function. A drop in the *Q* factor from 13,000 to 8,000 is observed indicating relatively weak mR coupling to the QTF compared to the on-beam configuration, where the *Q* changes from 13,000 to as low as 1,380. A maximum off-beam-QEPAS signal was obtained when the mR slit was positioned 1 to 1.5 mm below the QTF opening, as observed in the case of the on-axis QEPAS configuration (see Figure 8). In [57] Liu *et al.* performed an experimental investigation of the dependence of the OB-QEPAS signal as a function of the mR length and inner diameter. They observed that the inner diameter-to-optical length ratio linearly increases with the inner diameter. Once the inner diameter is chosen for a specific application, the optimal ratio can be determined. In addition, for efficient coupling of the acoustic signal from the mR to the QTF via a slit, it is also necessary to optimize the slit size. A small slit size limits the coupling of the acoustic energy, while a large slit size disperses the output sound energy. Varying the tube length shifts the resonant frequency of the mR and the QTF operates as a fixed-frequency probe. By using the theory of finger-holes in woodwind instruments it is possible to find a relation between the tube length and the mR resonance. The off-beam-QEPAS signal as a function of the resonant frequency calculated for each mR length exhibits a Lorentzian profile, in good agreement with a classical driven oscillator [38], centered at *f* = 32.11 kHz with a FWHM = 5.78 kHz corresponding to a *Q* = 5.6. According to this theory, the tube length corresponding to *f_0_* = 32,750 Hz is *l* = 7.56 mm. Liu *et al.* found experimentally an optimal slit width of ∼ 0.15 mm with a length of ∼ 0.4 mm, for a mR with an outer diameter of 0.7 mm, an inner diameter of 0.45 mm and a length of 8 mm [55]. For these optimal conditions, a photoacoustic signal of ∼15.7 times higher than that corresponding to a QEPAS system using a bare QTF is obtained.

The SNR is reduced by a factor of ∼1.7 at atmospheric pressure for off-beam-QEPAS as compared to the on-beam design. However, off-beam-QEPAS is more flexible in terms of the QTF geometry employing custom-made QTFs, with a smaller gap between the prongs without complications related to using an optimized excitation laser pump beam. An off-beam-QEPAS spectrophone is also technologically easier to assemble and align.

A low-cost UV-LED (compared to the diode lasers and QCLs) has been recently employed as light source in an off-beam-QEPAS setup for ozone detection and a detection limit of 1.27 ppm corresponding to a normalized noise equivalent absorption (NNEA) parameter of 3.02 × 10^−8^ cm^−1^W/Hz^−1/2^ was achieved [58,59].

### 3.4. Fiber-coupled QCL-QEPAS

Enhanced versatility of QEPAS sensor systems in terms of flexible laser beam guidance and compactness can be obtained with optical fiber delivery of the laser source. Compact sensors with simple optical alignment have been realized by employing a single mode fiber delivery system of a near-IR laser source and a QEPAS ADM. The extension of this approach to mid-IR QEPAS sensors is limited by the lack of low-loss, single-mode optical fibers. A single spatial mode laser beam is mandatory for QEPAS sensing since the radiation blocked by the ADM creates an undesirable background that is usually several times larger than the QTF thermal noise level, and is accompanied by a shifting fringe-like interference pattern, which limits the detection sensitivity [37,60].

Both solid core and hollow core waveguides (HCWs) have been demonstrated to be very efficient for mid-IR QCL beam single-mode delivery [61,62]. However solid core fiber operation becomes multi-mode at wavelength λ > 3 μm and in this range HCWs represent the only solution. In HCWs the laser beam propagates through an air core by multiple reflections on a metallic inner wall. The main advantages are a high power threshold, low insertion loss, no-end reflections and low beam divergence at the waveguide exit. In addition, the waveguide core is coated with a dielectric film with a thickness suitably chosen to minimize waveguide transmission losses in the metallic layer at the wavelength of the propagating laser radiation [63].

**Figure 10.**
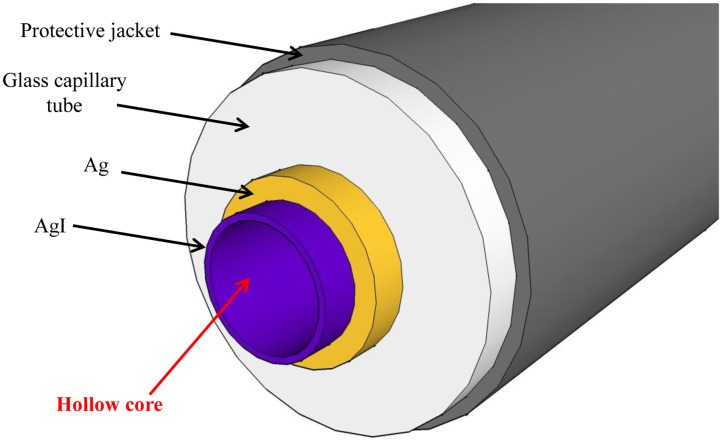
Simple schematic of the hollow fiber with Ag/AgI coating. Thicknesses are not drawn to scale.

Recently, Spagnolo *et al.* have developed a QEPAS sensor system using a HCW coupled single mode QCL pump source. Single mode laser delivery was obtained using a HCW with inner silver-silver iodine (Ag-AgI) coatings, an internal bore size of 300 μm, transmission losses of 1 dB/m and bending losses of 0.1 dB [64]. The basic structure of the fiber is shown in Figure 10.

**Figure 11.**
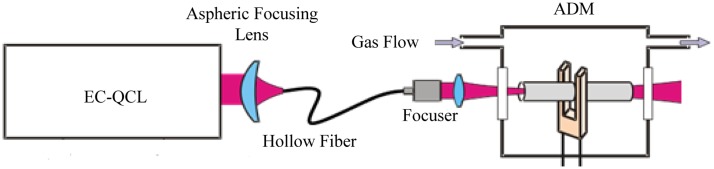
A fiber coupled EC-QCL based QEPAS sensor system.

To fabricate a hollow fiber for mid-IR applications, an Ag layer is deposited inside a glass capillary tube by flowing a silver solution through the tube. A dielectric layer is then subsequently formed by flowing an iodine solution through the same tube that reacts with the silver to form AgI. By controlling the thickness of the AgI dielectric layer, the transmission window of the fiber can be optimized for a specific wavelength range from 2.5 to 18 μm. The overall loss and spatial mode properties are mainly determined by the bore size. A single-mode Gaussian-like beam profile output can be obtained when d <30 λ. However, for practical applications a small bore size is not always desirable, since the smaller the bore size, the greater the loss, which scales as 1/d^3^. For laser wavelengths near λ∼10 μm, a 300 μm bore fiber offers a good compromise by providing single-mode beam delivery with only a moderate transmission loss. A fiber coupled QCL-QEPAS system is shown in Figure 11.

The EC-QCL output beam must be mode-matched to optimally couple into the ∼0.04 numerical aperture of the HCW. A ZnSe lens with a 12.7 mm diameter and 40 mm focal length was selected to ensure a focused spot diameter of <300 μm in order to maximize the laser light delivered by the HWG to the QTF. A beam waist diameter of ∼160 μm was measured in the focal plane of the focusing lens, which is well below the gap width of the QTF prongs. Hence, 99.4% of the laser light exiting from the collimator was transmitted through the ADM without touching it and allowing a strong reduction of the background finger-like pattern in QEPAS spectra [65]. The fiber coupled QCL-QEPAS system was then tested by employing a 10.54 μm EC-QCL with an output power of up to 90 mW, and choosing SF_6_ as the target gas. A SF_6_ absorption line located at 948.615 cm^−1^ was selected, which is interference-free from water interference. For a 1 s lock-in integration time a minimum detection sensitivity of 50 ppt was achieved, corresponding to a NNEA = 2.7 × 10^−10^ cm^−1^ W Hz^−1/2^, which represents a record value for the QEPAS technique [66].

### 3.5. MOCAM Technique Combined with QEPAS

The modulation cancellation method (MOCAM) is a variation of modulation spectroscopy using two light sources [67]. The basic concept of MOCAM is that the powers and modulation phases of two light sources can be adjusted to balance out the background signal, which limits the accuracy of measurements. In order to perform this technique, the output powers and modulation phases of the two light sources must be tuned to be in resonance with two selected gas absorption lines, which can be adjusted so that the signal detected from the reference sample is zero. In this case, the signal from the analyzed sample will be directly proportional to the deviation of the absorption line strength ratio from the reference line strength ratio. MOCAM can be used in various implementations that include WM (*1f* or *2f* detection) absorption spectroscopy. For example, the MOCAM technique combined with QEPAS was successfully used for measurements of temperature differences in a gas mixture [68–71], of isotopic composition [72,73] and for the detection of broadband absorbers at atmospheric pressure [69–71].

#### 3.5.1. Temperature Measurements and Isotopic Composition of a Gas Mixture

**Figure 12.**
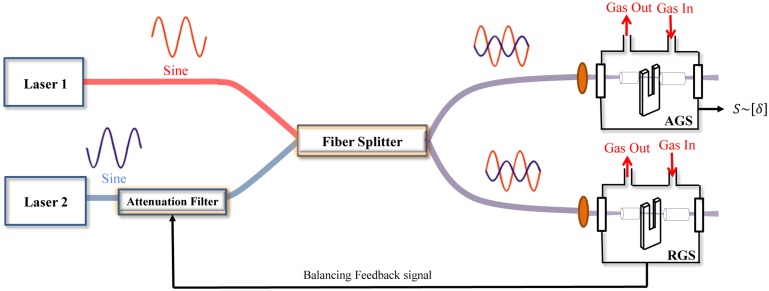
MOCAM setup for isotope concentration ratios and temperature measurements. AGS – Analyzed gas sample, RGS – Reference gas sample.

A schematic of the MOCAM-QEPAS architecture for isotopic composition and temperature measurements is shown in Figure 12.

Two QCLs are wavelength-modulated with the same frequency and the combined emission is directed to two QEPAS cells, one containing the reference gas sample and the other the analyzed gas sample. The central wavelength of both lasers must be locked to two selected absorption lines of the two isotopes gas target (in case of temperature measurements the pair of optical transitions belong to the same molecular species). The modulation phase and optical power are manually set in such a way that the QEPAS signals at *2f* produced by the two lasers are identical and opposite in phase. The phase relations are maintained by a phase locked loop. In other words, the two QEPAS signals in the reference cell can be balanced in such a way that no generation of sound occurs and the QTF detects no signal with an uncertainty equal to the QTF thermal noise level. For this condition, the signal from the analyzed sample will be directly proportional to the deviation of the absorption line strength ratio from the reference ratio in the selected optical configuration.

Spectroscopic temperature measurements are based on the temperature dependence of the absorption line strength ratio (but independent of pressure) for a pair of optical transitions of the same chemical species with different lower energy levels. The principle is based on the selection of two spectroscopic transitions originating at lower states with different energy, can be expressed as follows [68]:

(27) $$R=\frac{S_{1}(T_{0})}{S_{2}(T_{0})}e^{-\frac{hc\Delta E}{k}\left(\frac{1}{T}-\frac{1}{T_{0}}\right)}$$

where *S_1_* and *S_2_* are the absorption linestrength of two selected lines, *c* is the speed of the light, *h* the Planck constant and *k* the Boltzmann constant. The exponential terms contains the difference in lower state energy *ΔE* between the two absorption lines accounts for the Boltzmann populations at temperatures *T* and *T_0_*. In a first approximation for small changes of temperature, R is proportional to:

(28) $$R\propto e^{-\frac{\Delta E}{T}}$$

Here both *E* and *T* are expressed in energy units for simplicity. Thus:

(29) $$dT\propto \frac{dR}{R}\frac{T^{2}}{\Delta E}$$

Since *T* and *ΔE* can be assumed approximately constant, the precision in measuring deviation *dT* of the analyzed sample temperature from the reference sample temperature is dominated by the error in measuring *dR/R*. Assuming that the QEPAS spectrophones are similar and the temperature difference is small compared to the absolute temperature, it is possible to write:

(30) $$\frac{\Delta R}{R}\propto \frac{1}{U_{1}}(U\pm \delta \sqrt{2})$$

where *U_1_* is the signal detected from the analyzed sample when laser 2 (or its modulation) is turned off and *U* is the signal detected from the analyzed sample when the signal from the reference cell is zero, cancelled by balancing the two lasers. The $\sqrt{2}$ coefficient reflects the fact that the noise of two spectrophones is uncorrelated and, therefore, adds in quadrature. *δ* is the deviation from the reference and can be expressed as:

(31) $$\delta \left[\%\right]\propto \frac{R-R_{st}}{R_{st}}100$$

where *R_st_* is the ratio in the reference sample. Since the temperature difference between two samples will be calculated using Equation (29) and *ΔR/R* is obtained from Equation (30), the temperature difference is:

(32) $$\Delta T=C_{M}\frac{U}{U_{1}}$$

*C_M_* is a constant, which include the term *T^2^/ΔE*, which depends on the MOCAM configuration and is determined by an initial setup calibration. The uncertainty *δT* in the *T* measurements due to the noise in the photoacoustic signal *δ* is given by [67]:

(33) $$\delta T=\frac{R}{\frac{dR}{dT}}\frac{\delta \sqrt{2}}{U_{1}}$$

A C_2_H_2_/N_2_ gas mixture with 0.5% C_2_H_2_ concentration was used as a test analyte. The achieved sensitivity was 30 mK with a lock-in integration time of 17 s [68].

The MOCAM technique combining QEPAS with *2f* wavelength modulation was also used to measure the isotopic ^18^O/^16^O ratio in water vapor. The main difference is that in this case two spectral lines were selected for the two different water isotopes, *i.e.*, H_2_^16^O and H_2_^18^O. In particular, the selection of the isotope absorption lines required that the related lower energy levels were close with the same quantum numbers, in order to limit the sensitivity of the measurement to temperature variations and to guarantee identical broadening and relaxation properties [72]. The sensitivity reached in measuring the deviation (expressed in the δ-notation as a deviation from the reference ratio) from a standard sample δ^18^O was 1.4‰ for a 200 s lock-in integration time [72,73].

#### 3.5.2. Detection of Broadband, Polyatomic Molecules

Vibrational spectra of most molecules consisting of more than five atoms are dense so that Doppler and pressure broadening make them unresolved at normal temperature and pressure conditions. As a result, infrared absorption spectra of such polyatomic molecules consist of broad spectral bands, that are 100–200 cm^−1^ wide. Spectroscopic identification of these species requires optical sources with wide spectral coverage, Thus, QEPAS detection of such molecules requires amplitude modulation of the laser radiation, which is not recommended for sensitive concentration measurements due to the presence of a coherent background generated by the scattered and subsequently absorbed light.

**Figure 13.**
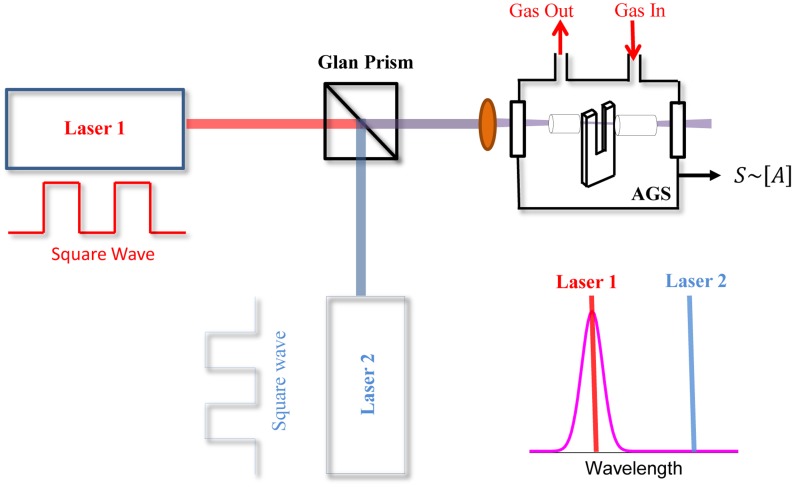
MOCAM method applied to the detection of broadband absorbers such as polyatomic molecules and aerosols. AGS – Analyzed gas sample. The signal S will be proportional to the target gas species concentration ([A]).

However, MOCAM technique combined with QEPAS detection offers an interesting alternative for sensitive detection of broadband absorbers in the presence of spectrally nonselective background absorption [69–71]. The schematic of a MOCAM based sensor architecture for broadband molecule detection is shown in Figure 13.

This approach can be used for the detection of species with broad unresolved absorption features such as the majority of polyatomic molecules. In this case, one of the lasers is centered on the target absorption band while the second source is tuned to the background region. When the lasers 1 and 2 are amplitude modulated with 180° phase shift, then the modulation amplitudes can be adjusted so that there is no signal in the absence of *A*. If the concentration *[A] ≠0*, the signal detected at the modulation frequency is proportional to the *A* concentration. For example, MOCAM has been successfully used for the detection of hydrazine (N_2_H_4_), a highly toxic, unstable, strongly corrosive substance, used in polymer production, the pharmaceutical industry, and as a rocket fuel. Two independent wide stripe Fabry-Perot diode lasers were used to detect near-IR absorption of hydrazine vapor, with emission covering both the tail and the peak of the N_2_H_4_ absorption band. Both lasers 1 and 2 emit linearly polarized light. The polarization plane of one of the lasers was rotated 90° by means of half-wavelength plate (not shown in Figure 13). The radiation emitted by the two lasers was combined using the Glan polarizer. The overlapped images of the laser facets were located between the QTF prongs. No acoustic micro-resonator was used, which reduced the probed optical path to the QTF thickness, 0.3 mm [69–71]. The MOCAM technique suppressed the background signal level by ∼ three orders of magnitude as compared to unbalanced (one laser) detection. The hydrazine vapor detection limit was ∼ 1 ppm with a 1 sec averaging time, corresponding to 4.1 × 10^−9^ fractional absorption in the probed 0.3 mm path length. Noise suppression and sensitivity did not improve in the balanced detection mode and was laser-dominated, ∼100 times higher than the QTF thermal noise limit [69–71].

Wide stripe diode lasers are usually not used for spectroscopic applications because they exhibit high divergence and multimode (both spatially and spectrally) behavior. Therefore, there is no way to efficiently couple the radiation from a wide stripe laser into a multipass cell or an optical cavity. However, this is possible for the QEPAS technique because QTFs are wavelength insensitive transducers with a short optical path length (∼0.2–0.4 mm, *i.e.*, the QTF thickness).

### 3.6. Quartz-Enhanced Evanescent-Wave PAS

In all PAS system reported so far, open-path cells have been used and precise collimating/focusing optics or fiber-coupling systems minimize the optical insertion loss and improve the efficiency of photoacoustic signal generation. An alternative is the use of tapered optical micro/nano fibers to generate evanescent waves for the photoacoustic generation.

**Figure 14.**
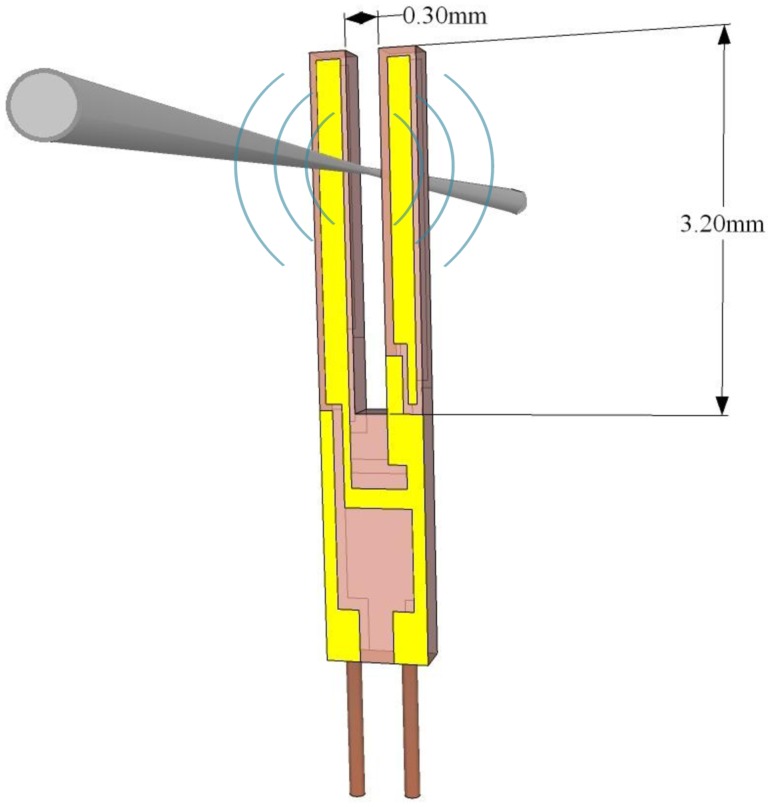
Schematic of the quartz-enhanced evanescent-wave PAS configuration with a tapered optical micro fiber. The green circular lines represent the wavefronts of the acoustic wave.

Fiber tapers with diameters down to the sub-wavelength scale and low loss of ∼0.2 dB can be fabricated by using a flame-brushing technique starting from standard single-mode fibers and controlling the flame movement and fiber stretching rate during processing [74]. The tapered fiber is threaded through the gap between the two prongs of the QTF, and thus the laser radiation is guided along the fiber with a very small beam size, hence no precise optical alignment is needed. Wavelength-modulated light from a single-mode near-IR diode laser is transmitted to the tapered fiber, and the evanescent field is absorbed by the nearby target gas, generating an acoustic pressure wave that is detected by the QTF. The quartz-enhanced evanescent-wave PAS configuration is schematically shown in Figure 14.

With a fiber taper of 1.1 μm waist diameter, a DFB laser with wavelength of 1,532.8 nm and 9.8 mW optical power used as the light source, and a standard QTF (*f_0_* = 32,768 Hz), a minimum detectable concentration limit of 178 ppm was achieved for C_2_H_2_ detection at atmospheric pressure (with a lock-in integration time of 30 ms), corresponding to a NNEA = 1.96 × 10^−6^ cm^−1^W/Hz^1/2^ [75]. This detection sensitivity can be further improved by reducing the diameter of the fiber taper and by using a custom-made QTF optimized for this purpose. Calculations based on finite element method have shown that as fiber diameter is reduced to <1 μm, the percentage of evanescent field in air increases rapidly. When the diameter of fiber is 0.6 μm, >80% of the light power will be in the evanescent field, which enables efficient interaction between sensing gas and laser source [76]. This method provides an alternative to the general open path-based QEPAS, and has the potential advantages of lower insertion loss, easier optical alignment, and a potentially multiplexed sensing capability.

## 4. Terahertz QEPAS

### 4.1. Terahertz Spectroscopy for Gas Sensing

Terahertz (THz) radiation lies in the frequency gap between the infrared and microwaves and has been studied in various science and engineering fields, such as astronomy and analytical science. Milestones of THz research include the development of THz time-domain spectroscopy, THz imaging and THz generation by means of non-linear effects [77,78]. In the last ten years, photonics has led to the realization and the development of THz QCLs providing compact and stable sources with high power, continuous-wave operation and single-mode emission. Nanotechnology research has demonstrated the possibility to use semiconductor nanowire as low-noise and room temperature THz detectors based on a field-effect transistor configuration, offering an useful alternative to bulky and costly cryogenic bolometers [79]. Due to recent innovations in photonics and nanotechnology, THz research can be applied in a wide variety of applications, including information and communications technology, medical sciences, homeland security, quality and industrial process control [80]. Explosives, narcotics and toxic gases have spectral fingerprints and strong absorption bands in the THz spectral range.

The energy of photons in the THz region allows the study of vibrational activity outside the range of infrared spectrometers, and rotational and torsional modes of gaseous molecules of higher energy than those observed by microwave spectrometers. THz QCLs have recently demonstrated good performances in high-resolution molecular spectroscopy [81–85]. Many gases have absorption strengths that are of the same order of magnitude as those observed in the mid-IR spectral range and three to six orders of magnitude stronger than those measured in the microwave region. For example, gas species such as water, nitrogen compounds, oxygen and chlorine possess strong absorption bands in the THz range and the precise knowledge of their concentration in the stratosphere and in the upper troposphere is important for the study and monitoring of chemical processes related to ozone depletion, pollution monitoring and global warming. THz and sub-THz radiation has long been a valuable tool for astronomers in characterizing astronomical systems due to the ability of microwave and THz spectra to identify gases by means of their rotational spectra. High-resolution rotational spectroscopy is also invaluable in providing very precise structural information regarding molecular species. Rotational spectroscopy is a well-developed and documented area: extensive libraries of rotational spectra and texts on this subject exist [86].

### 4.2. Extension of QEPAS Technique in THz Range: Custom-Made Tuning Fork

The QEPAS technique is also an excellent candidate for high performance THz gas spectroscopic techniques for two reasons: no optical detection is required and the QEPAS signal strongly depends on the energy relaxation rates of the absorbing gas species. In gas absorption processes that occur in the THz range, rotational-translation (R-T) relaxation rates are involved, and it was demonstrated that R-T relaxation rates are up to three orders of magnitude faster with respect to V-T rates commonly involved in the mid-IR absorption [87]. Thus, the possibility to work with fast relaxing transition levels allows operating at low gas pressures, and taking advantages of the high QTF Q-factor enhancing the selectivity of a QEPAS sensor system. The extension of the QEPAS technique to the THz spectral region was delayed, mainly due to the difficulty of a proper focalization of the THz laser beam between the prongs of the QTF, since (i) the gap between the prongs is comparable with the wavelength of the THz radiation and (ii) a THz beam is characterized by a lower spatial radiation quality. As discussed in previous sections, one of the main issues in mid-infrared QEPAS based sensor systems is due to the required optimum focusing between the QTF prongs. The laser beam must not touch the prongs since otherwise an undesirable non-zero background arises due to the laser contribution. Hence, the use of QTFs with larger prong spacing is mandatory to extend QEPAS operation in the THz range.

**Figure 15.**
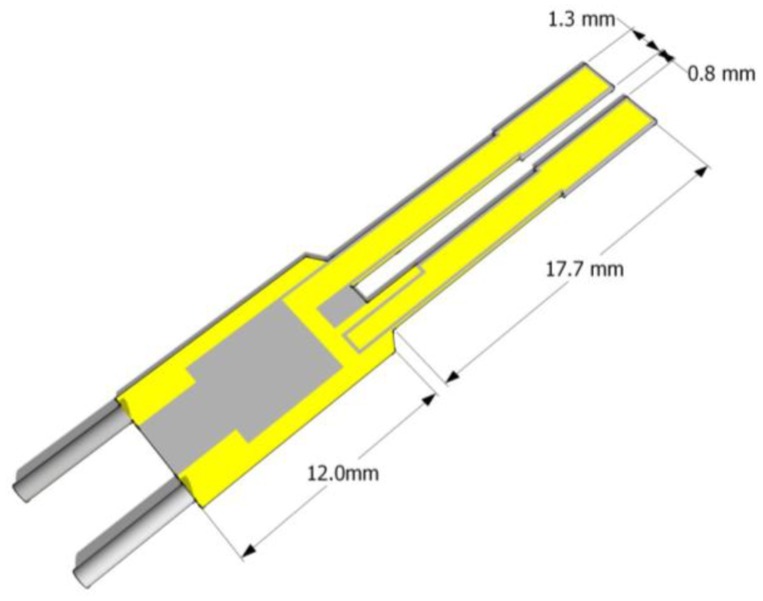
Schematic of a THz quartz crystal tuning-fork.

Recently a 4 kHz QTF (T-QTF) having the same geometry as the standard 37.8 KHz, but with a 6 times bigger gap spacing was reported [88,89]. T-QTFs have been realized by using standard photolithographic technique to etch z-cut quartz wafer. Electrical contacts are designed by depositing 600/2,000 Å chromium/gold layers on both sides of the T-QTF [90]. The T-QTF dimensions are 3.3 cm × 0.4 cm, with a thickness of 0.8 mm; each prong is 17.7 mm long and 1.3 mm wide. The prongs are separated by a gap of ∼800 μm. The schematic of the T-QTF resonator is shown in Figure 15.

By inserting the dimensions of the T-QTF in Equation (12), it is possible to estimate the first two resonance frequencies related to the two flexural vibrating mode in the vacuum: 4,118 Hz and 25,786 Hz. Resonance frequencies of *f*_1_= 4.245 kHz and *f*_3_ = 25.4 kHz were measured by using the CEU, at atmospheric pressure, and the corresponding quality factors are: Q_1_ = 13,100 and Q_3_ = 9,800. The discrepancies from the theoretical values can be attributed to four factors: (i) damping effects of the ambient gas; (ii) additional weight of the electrode gold layers; (iii) dependence of the elasticity modulus of quartz on the crystallographic axes orientation; and iv) deviations in geometry between the modeled and the real T-QTF. To study the damping effects induced by the environmental gas the resonant frequencies and the quality factor dependences as a function of the gas were investigated. As theoretically predicted in Equation (14), both resonance frequencies have shown a linear dependence for the gas pressure in the investigated range 10-780 Torr. The *Q* factor pressure dependence shows an exponential behavior, as predicted in Equation (17), and rapidly decreases with the gas pressure. The results obtained from the T-QTF characterization demonstrated that a T-QTF behaves like a near-IR and mid-IR QTF [89].

**Figure 16.**
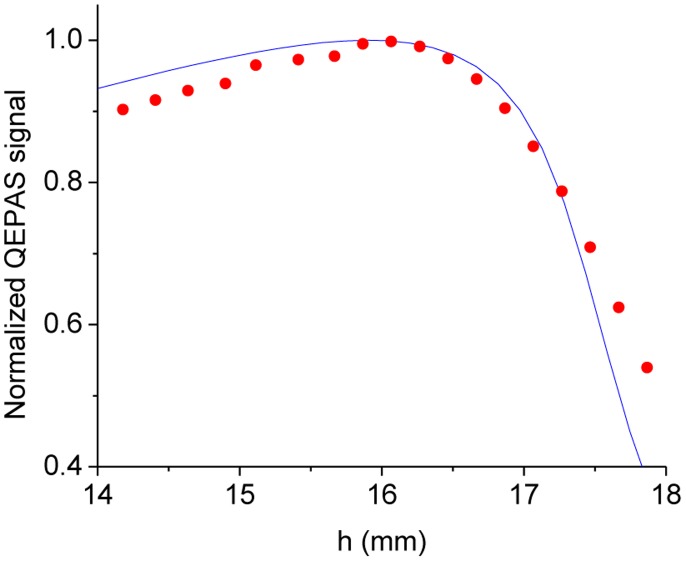
Normalized experimental results (dots) of the QEPAS signals as a function of the vertical position of T-QTF. Data are obtained by detecting 1.55% of methanol in N_2_ at 10 Torr and by using the experimental setup depicted in Figure 17. The solid line is the related best fit curve using Equation (3.21).

Figure 16 shows the dependence of the QEPAS signal as a function of the vertical position of the laser beam and the best fit obtained by using Equation (25), in order to obtain the vertical position that maximize the peak value of the THz QEPAS signal. Good agreement between experimental data and calculation is observed, which confirms the validity of our model.

### 4.3. THz QEPAS sensor for Methanol Detection

**Figure 17.**
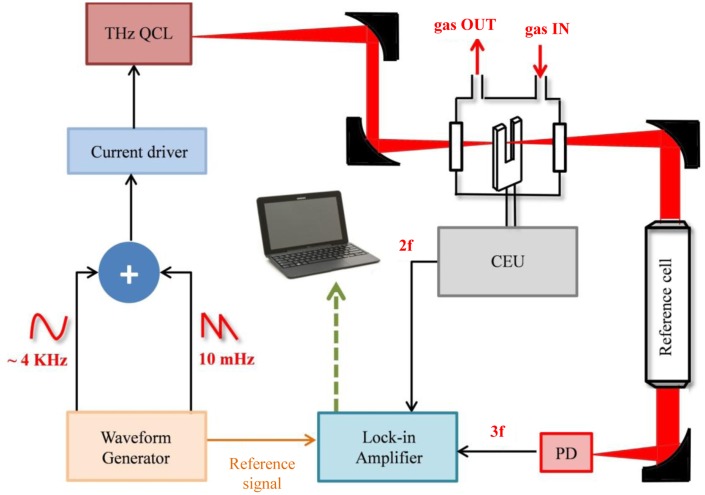
Schematic of a THz QEPAS based trace gas sensor. PD – Photodetector.

A schematic of the THz QCL-based QEPAS sensor is shown in Figure 17. The THz laser source is a single-mode CW THz QCL emitting at ∼76.3 μm (3.93 THz) [91] mounted on the cold finger of a continuous-flow cryostat equipped with polymethylpentene (TPX) windows and operating at T = 6 K. The THz beam was focused between the two prongs of the tuning fork by using two 90° off-axis paraboloidal gold reflectors. The QTF is housed in a cell with TPX input and output windows. The laser beam was re-collimated upon exiting the cell by means of a gold parabolic mirror. A reference cell (15 cm-long) was filled with high concentration of the gas species under study for spectral reference. Another gold parabolic mirror was used to focus the laser beam exiting the reference cell to a pyroelectric detector. By means of a pyroelectric camera; a focused beam waist of ∼430 μm between the QTF prongs was measured, which is well below the gap separation of ∼800 μm between the QTF prongs.

For a THz-QEPAS spectral scan, the laser frequency can be scanned over 0.025 cm^−1^ by applying a low-frequency (10 mHz) voltage ramp up to 1 V to the external analog modulation input of the QCL current supply. The THz laser output power was ∼180 μW. A sinusoidal dither at frequency f_1_ is simultaneously added to the low-frequency voltage ramp, to obtain optical frequency modulation of up to 0.01 cm^−1^.

The selected methanol absorption line was the rotational translational transition located at ν_line_ = 3.9289 THz (131.054 cm^−1^) with a line-strength S = 4.28 × 10^−21^ cm/mol in HITRAN units [92]. Gas mixtures with different methanol concentrations in pure N_2_ were obtained by diluting methanol vapor, collected from a reservoir held at a vapor pressure of 120 Torr at 300 K, with pressurized N_2_. For measurements at low concentrations, a certified 100 ppm methanol/N_2_ gas mixture was used. The optimal sensor operating conditions were found to occur by using the first resonant frequency *f_1_* of the T-QTF, at 10 Torr and a modulation amplitude of 600 mV. The linearity and detection sensitivity of the QEPAS sensor were evaluated by measuring its response to varying methanol concentration in pure N_2_. For a 4 s averaging time (and a bandwidth of 0.04169 Hz) a detection sensitivity of 7 ppm, corresponding to a NNEA of 2.7 × 10^−1^ cm^−1^ W/Hz was obtained [88,89]. This value is comparable with the best results obtained in the mid-IR spectral range [64–66] and comparable with the detection sensitivity achieved with cryogenic bolometers.

THz QEPAS detection limit can be further improved by: (i) employing a THz QCL with higher output power (>100 mW has been reported in [93]); (ii) selecting molecules such as HF, H_2_S, OH, NH_3_, and HCN, having absorption strengths larger than 10^−19^ cm/mol; and iii) a custom designed T-QTF of improved geometry in terms of sensing performance.

## 5. Review of QEPAS-Based Trace Gas Detection

Minimum detection limits can be quantified as the noise equivalent concentration (NEC) or the minimum detectable absorption coefficient (α_min_, in cm^−1^), allowing different sensors to be compared without reference to the specific target gas. For estimates of noise and precision, we use the convention that the noise equivalent concentration (NEC) is the gas target concentration giving a signal equal to the root mean squared (RMS) value of signal intensity variations (1σ). Although the QTF thermal noise represents the physical limit for sensor detection, in practice it is not easy to reach such a low noise level (∼μVs). Therefore, for many sensor systems, white noise dominates and SNR depends on the bandwidth Δf, as SNR is ∝ Δf ^−1/2^. Furthermore, it is important to also record the value of the measurement integration time *t* used to obtain a certain noise limit, and*/*or to quote limits in units of normalized noise equivalent absorption coefficient (NNEA) measured in cm^−1^W/Hz^−1/2^, by normalizing the noise equivalent absorption to a 1 Hz measurement bandwidth.

In Table 2 we listed the results obtained so far for QEPAS based gas sensors. For each gas target, we reported the operating spectral region and pressure, the available laser optical power at the input to ADM, the type of QEPAS system and the sensor performance in terms of NEC and NNEA.

NNEAs measured to date using QEPAS are better than the best conventional PAS results. A record NNEA of 2.7 × 10^−10^ cm^−1^W/Hz^1/2^ is obtained for SF_6_ detection at a gas pressure of 75 Torr, employing an external cavity mid-IR QCL fiber coupled to the spectrophone [66]. The sensitivity of the sensor is a result also of exceptionally large SF_6_ absorption cross-sections and its fast V-T relaxation. CO detection in N_2_ is characterized by a low NNEA (5.3 × 10^−7^ cm^−1^W/Hz^1/2^) confirming that the V-T relaxation of the CO fundamental vibration is slow for efficient acoustic generation at 32.7 KHz [29]. A propylene host was found to promote the V-T relaxation of CO leading to an improvement of the NNEA of ∼ one order of magnitude [30]. Similarly, also N_2_O is characterized by a slow V-T relaxation and in this case SF_6_ was added to the gas sample to promote the V-T relaxation leading to a N_2_O NEC of 7 ppb, for a 1 s lock-in integration time [29]. For CO_2_ detection, the influence of the H_2_O presence as a relaxation promoter in the sample gas mixture was investigated in considerable detail. Water vapor is known to be an efficient catalyst for the vibrational states. Therefore increasing the water concentration in gas mixtures involving molecules with slow V-T relaxation rates increase the detected CO_2_ QEPAS signal amplitude, up to a 1.5% of H_2_O concentration, leading to a minimum detection limit of 18 ppm for a 1 s lock-in integration time [31]. Higher H_2_O concentrations result in a lower QEAPS signal, since the relaxation process of CO_2_ molecules are dominated by CO_2_-H_2_O collisions. Similarly, the QEPAS-based NO detection can be improved by a factor of almost 50 by the addition of H_2_O with respect to dry NO in N_2_ [37]. Furthermore, from the results in Table 2 the optimum pressure range to detect molecules with isolated fast-relaxing transitions is ∼50–90 Torr.

**Figure 18.**
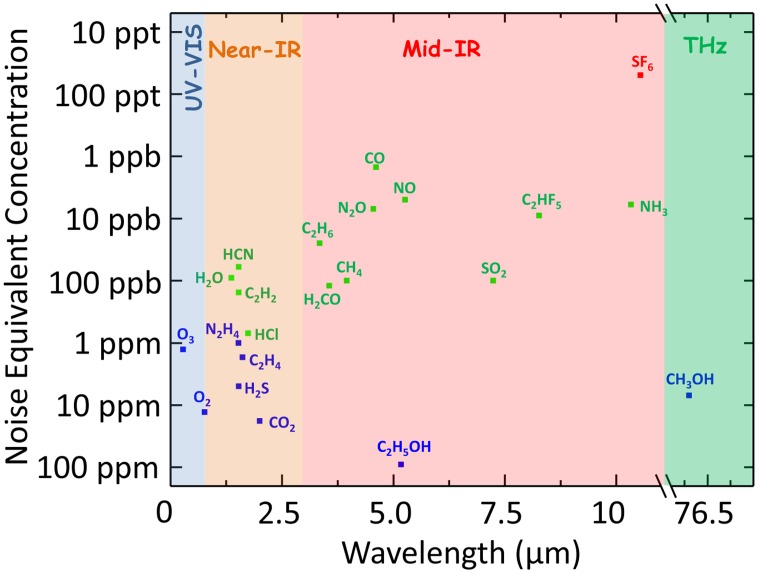
Best NEC results for the gases listed in Table 2 *versus* employed laser wavelength, in the UV-Vis, near-IR, mid-IR and THz spectral ranges. The blue, the green and the red symbols indicate NEC values in the ppm, ppb and ppt concentration ranges, respectively.

In Figure 18 the best noise equivalent concentration are plotted for all investigated gases depicted in Table 2 as a function of the employed laser wavelength. Since the NEC is related to the laser power and the absorption linestrength, the best results have been obtained in the mid-IR fingerprint range where powerful QCLs are available. The low NEC value reported for ethanol [34] is due to large spectral absorption feature that is a characteristic of this gas and which prevents the use of the efficient WM QEPAS approach and permits only the use of the AM QEPAS method.

### 5.1. Long Term Stability of a QEPAS Sensor

Measurements are required to characterize long-term drifts and establish the signal averaging limits, while the NNEA describes the sensor performance on a short time scale. The approach, described in [97] introduces the Allan variance of time sequences of measurements to quantify the long-term stability of optical trace gas sensors. The laser radiation was locked to the absorption line peak while the absorbing gas concentration and the gas pressure are maintained constant. For these conditions, by fixing the lock-in amplifier time constant, the lock-in amplifier readings are recorded, by using an acquisition time at least three-fold the lock-in time constant. The measurements were performed continuously for several hours. To perform an Allan variance σ_A_ analysis, all the data subsets with the laser targeted onto a specific absorption line have to be stacked together and treated as a single uninterrupted time sequence. Usually the Allan deviation is shown instead of the variance and expressed in terms of absorption coefficient or absorbing gas concentration for a particular sensor, thus allowing to determine the minimum detectable concentration as a function of the lock-in integration time. The Allan deviation $\sqrt{\langle {\sigma_{A}}^{2}\rangle }$ for all time sequences closely follows 1/√t dependence over the entire duration of the measurements series. This observation verifies that white Johnson noise of the QTF remains the dominant source of noise even on a multi-hour time scale, and that a QEPAS based sensor allows unlimited data averaging without base line or sensitivity drift [19,95]. However, for sensors operating in the field, after some optimum averaging time drift effects can be observed, mostly due to mechanical movements of different components comprising a QEPAS based sensor system. The point at which this occurs, and the extent to which performance deteriorates thereafter, are both application– and installation– specific for a given sensor instrument.

## 6. Comparison with Existing Optical Techniques and Perspective

**Figure 19.**
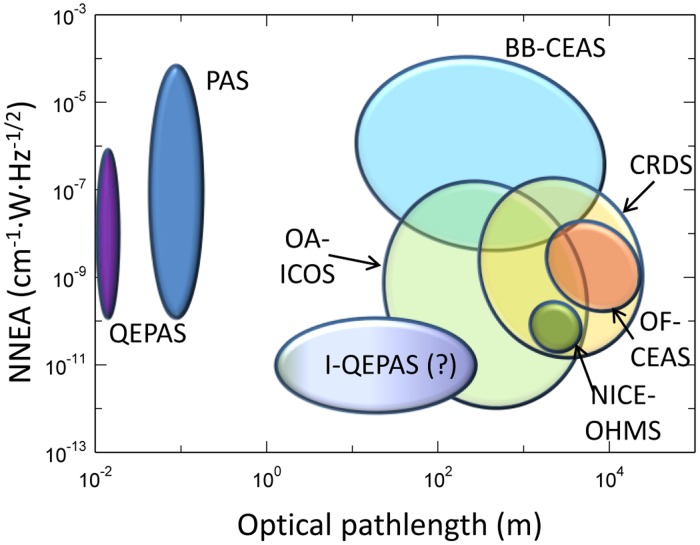
NNEA for categories of gas detection techniques as a function of optical path-length. Key: BB-CEAS – broadband cavity-enhanced spectroscopy, CRDS – cavity ring-down spectroscopy, OA-ICOS – off-axis integrated cavity output spectroscopy, OF-CEAS – optical feedback cavity-enhanced absorption spectroscopy, NICE-OHMS – noise-immune cavity-enhanced optical heterodyne spectroscopy, PAS photoacoustic spectroscopy, QEPAS – Quartz-enhanced photoacoustic spectroscopy, and I-QEPAS – Intracavity quartz-enhanced photoacoustic spectroscopy.

When comparing different techniques a figure of merit that makes sense in one application is of limited value in another, or even difficult to calculate. A possible common metric to compare the performances of different types of laser based sensors should take into account the available optical laser power, the strength of the selected absorption line and the integration time used in the measurements. In this case, the normalized noise equivalent absorption parameter, when it is possible to compute, represents the best choice. In Figure 19 we compared the performance of several gas detection techniques in terms of NNEA *versus* optical pathlength [98,99]. It should be noted that in most cases the reported NNEA was obtained in a laboratory setting and such performances cannot easily be replicated in actual field environment. For example, it can be challenging to maintain the precise alignment needed in sensor systems with long equivalent optical pathlengths.

The techniques with the lowest *NNEA*, *i.e.*, those capable to reach ppb and ppt gas detection concentrations, are those characterized by very long pathlengths (up to tens of km), namely CDRS, ICOS and CEAS. However, PAS and in particular QEPAS shows very good performance, reaching NNEA in the 10^−1^° cm^−1^ W/Hz^−1/2^ range, with the advantage of a much better sensor compactness.

Three main approaches have to be followed to realize optical sensors with high sensitivity: (i) selection of optimal molecular transition in terms of absorption strength and absence of possible interfering gases; (ii) long optical pathlength and/or cavity optical build up; (iii) efficient spectroscopic detection schemes. Since the QEPAS technique is characterized by direct proportionality between the signal amplitude and the laser power available for gas excitation, the higher the optical power focused between the QTF prongs, the lower will be the QEPAS sensor minimum detection limit. Thus, the possibility to realize intracavity optical build-up and QEPAS detection sensor design may lead to the realization of an optical sensor system with unprecedented detection sensitivity. Recently a cavity-enhanced optical feedback-assisted PAS sensor was demonstrated for water vapor detection reaching a noise-equivalent absorption value of 1.9 × 10^−10^ cm^−1^Hz^−1/2^ [100]. Furthermore, the development of an innovative spectroscopic technique, which we called intracavity QEPAS (I-QEPAS) was reported in [101]. This technique can be considered as a merging of CEAS and QEPAS for which we estimate an optical resonator enhancement factor of ∼250 and an optical pathlength of 84 m. The I-QEPAS method has been used to detect CO_2_, reaching a sensitivity of 230 ppt with 10 s averaging time and a corresponding NNEA of 2.5 × 10^−10^ Wcm^−1^/Hz^1/2^ [101]. Since a bare QTF was used as an acoustic detection module, further improvement can be expected by adding a mR system to the ADM. An estimate of the I-QEPAS sensing capability can be obtained by starting from reported QEPAS record sensitivities, *i.e.* few tens of ppt detection and a corresponding NNEA in the 10^−10^ Wcm^−1^/Hz^1/2^ range [64–66] and reduce these values by the achieved resonator enhancement factor (∼250). This yields I-QEPAS sensitivities at ppq concentration levels and a corresponding record low NNEA value in the range 10^−0^ to 10^−12^ Wcm^−1^/Hz^1/2^, as schematically described in Figure 19.

## 7. Conclusions

This work has focused on recent advances in the development of quartz-enhanced photoacoustic trace gas sensors for detection, quantification and monitoring of trace gas species. The current availability of commercial and research quantum cascade laser sources has allowed the targeting of strong fundamental rotational-vibrational gas absorption lines in the mid-IR spectral range and pure rotational lines in the THz range, that are one to two orders of magnitude stronger than overtone transitions in the near infrared. Progress to-date in terms of spectrophone configuration, performance optimization and sensitivity for QEPAS sensors was reviewed. The best results in terms of minimum detectable gas concentration have been obtained by employing acoustic micro-resonator tubes and positioning a commercial, low cost 32.78 kHz QTF between the tubes to probe the acoustic vibration excited in the gas contained inside the tubes (on-beam configuration). This configuration increases the QEPAS sensitivity up to 30 times compared with a QEPAS sensor with a bare QTF and ensures excellent acoustic isolation from accidental external resonances in the sensor enclosure. Moreover, the optical alignment is critical, especially with laser sources having limited spatial beam quality. To overcome these operating limitations three configurations have been developed. In the first approach, the tuning fork is placed close to a small slit made in the middle of a single resonator tube. The laser beam does not pass through the QTF prongs since it is located off-beam to acquire the photoacoustic signal. In the second approach, the laser beam is guided between the two prongs of a bare tuning fork (without resonator tubes) by using a tapered optical fiber and the associated evanescent-wave effect. These two approaches partially overcome and minimize the beam quality requirements associated with the on-beam configuration, but do not reach the same levels of detection sensitivity. The third approach is based on an optical coupling between the light source and a single-mode hollow core fiber combined with an on-beam configuration. The design of the source-fiber coupling and output collimator system achieved a beam waist diameter of ∼160 μm between the two QTF prongs, which simplified the optical alignment considerably. With such a configuration, the QEPAS sensitivity reached record detectable trace gas concentration levels in part-per-trillion range. QEPAS combined with a modulation cancellation technique was successfully used for measurements of temperature differences in a gas mixture, for isotopic measurements and for the detection of spectrally broadband polyatomic molecules. The recent development of long wavelength quantum cascade lasers made it feasible to use the QEPAS technique in the THz range by employing a custom-made QTF with a larger space separation between the two prongs to allow optimized THz radiation beam focusing, thereby reaching a detection sensitivity level comparable with the best results reported in the mid-IR. Moreover, an innovative spectroscopic technique, called I-QEPAS (a merging of CEAS and QEPAS methods) was recently demonstrated and potentially may lead to the realization of sensors with ppq detection limit and NNEA in the 10^−12^ Wcm^−1^/Hz^−1/2^ range.

In conclusion, compact, sensitive and selective QEPAS sensors have been demonstrated to be effective and mature for numerous real-world applications. These now include different fields such as environmental monitoring (CO, CO_2_, CH_4_, H_2_CO, C_2_HF_5_, N_2_O, NO_2_), industrial emission measurements such as at combustion sites and gas pipelines (HCl, CO_2_, CH_4_, CO, NO_x_, CH_2_O,), urban emission coming from automobile traffic (NO_x_, SO_x_), rural emission such as horticultural greenhouses and fruit storage (C_2_H_6_, C_2_H_4_, CH_4_, N_2_O), control for manufacturing processes (SF_6_, HCl), detection of medically important molecules (NO, CO, NH_3_, C_2_H_6_, H_2_S), toxic gases (CH_2_O, HCl, HCN, N_2_H_4_, *etc.*) and for planetary science (H_2_O, CH_4_,CO, CO_2_, N_2_H_4_, C_2_H_2_) and of course environmental monitoring For example a QEPAS-based sensor was installed in a mobile laboratory to perform atmospheric CH_4_ and N_2_O detection near two urban landfill sites located in Houston, TX [102]. The QEPAS sensor recorded concentration values in very good agreement (<5% difference) with those measured by the Aerodyne Research, Inc. “QCL mini monitor” multi-pass optical sensor having a CH_4_ detection sensitivity of 300 ppt and N_2_O detection sensitivity of 60 ppt, both in 1 s [103–105], which demonstrates the precision, stability and applicability of the QEPAS sensing technique.

## Figures and Tables

**Table 1.** n values and the resonant frequencies *f_n_* for the standard QTF fork calculated from Equation (3.8).

**n**	***f****_n_* **(Hz)**

1.194	31978
2.988	200263
5	560764

**Table 2.** QEPAS detection of trace gases using different QEPAS approaches. IT – signal integration time.

**Molecule (Target analyte)**	**Frequency cm^−1^**	**Pressure Torr**	**NNEA cm^−1^W/Hz^−1/2^**	**Power mW**	**NEC ppm**		**QEPAS system**		**IT (s)**	**Ref.**	
NH_3_ (exhaled air) *Ammonia*	6,528.76	90	8·10^−9^	7	5		On-beam		1	[26]	
CO_2_ (exhaled air) *Carbon dioxide*	6,514.25	90	10·10^−9^	5.2	890		On-beam		1	[26]	
H_2_O (exhaled air) *Water*	6,541.29	90	8·10^−9^	5.2	580		On-beam		1	[26]	
NH_3_ (N_2_) *Ammonia*	6,528.76	60	7.2·10^−9^	38	0.65		On-beam		1	[27]	
H_2_CO (air) *Formaldehyde*	2,832.48	200	2.2·10^−8^	3.4	0.55		On-beam		1	[28]	
N_2_O (air+5%SF_6_) *Nitrous oxide*	2,195.63	50	1.5·10^−8^	19	0.007		On-beam		1	[29]	
CO (N_2_) *Carbon monoxide*	2,196.66	50	5.3·10^−7^	13	0.5		On-beam		1	[29]	
CO (in propylene) *Carbon monoxide*	2,196.66	50	7.4·10^−8^	6.5	0.14		On-beam		1	[30]	
CO_2_ (air+1. 2%H_2_O) *Carbon dioxide*	4,991.26	50	1.4·10^−8^	4.4	18		On-beam		1	[31]	
HCN (air+50% RH) *Hydrogen cyanide*	6,539.11	60	4.6·10^−9^	50	0.155		On-beam		1	[32]	
CO_2_ (N_2_+1.5%H_2_O) *Carbon dioxide*	4,991.26	50	1.4·10^−8^	4.4	18		On-beam		1	[33]	
NH_3_ (N_2_) *Ammonia*	4,986.99	50	8.9·10^−9^	3.9	3		On-beam		1	[33]	
H_2_O (N_2_) *Water*	7,306.75	60	1.9·10^−9^	9.5	0.09		On-beam		1	[34]	
C_2_H_2_ (N_2_) *Acetylene*	6,529.17	75	2.5·10^−9^	40	0.06		On-beam		1	[34]	
CH_4_ (N_2_) *Methane*	6,057.09	950	2.9·10^−8^	14	2.1		On-beam		1	[35]	
H_2_CO (N_2_:75% RH) *Formaldehyde*	2,804.90	75	8.7·10^−9^	7.2	0.12		On-beam		1	[34]	
C_2_H_5_OH (N_2_) *Ethanol*	1,934.2	770	2.2·10^−7^	10	90		On-beam		1	[34]	
C_2_HF_5_ (Freon125) *Pentafluoroethane*	1,208.62	770	7.9·10^−9^	6.6	0.009		On-beam		1	[25]	
C_2_H_4_ (N_2_) *Ethylene*	6,177.07	770	5.4·10^−9^	15	1.7		On-beam		0.7	[94]	
H_2_O (N_2_) *Water*	7,165.82	770	1.68·10^−8^	8	9.27		On-beam		1	[36]	
NO (N_2_ humidified) *Nitric oxide*	1,900.08	250	7.5·10^−9^	100	0.005		On-beam		1	[60]	
NH_3_ (N_2_) *Ammonia*	6,528.76	575	3.3·10^−9^	25	0.06		On-beam		10	[38]	
H_2_S (N_2_) *Hydrogen sulfide*	6,357.63	780	5.6·10^−9^	45	5		On-beam		1	[39]	
CO_2_ (N_2_) *Carbon dioxide*	6,321.20	770	4.0·10^−9^	38	123		On-beam		1	[39]	
CH_4_ (N_2_+1.2%H_2_O) *Methane*	6,057.09	770	3.7·10^−9^	16	0.24		On-beam		1	[39]	
C_2_H_2_ (N_2_) *Acetylene*	6,529.17	770	3.3·10^−9^	37	0.085		On-beam		1	[40]	
NH_3_ (air humidified) *Ammonia*	967.35	130	/	24	0.006		On-beam		1	[41]	
CO_2_ (air humidified) *Carbon dioxide*	6,361.25	150	8.2·10^−9^	45	40		On-beam		1	[41]	
NH_3_ (N_2_+2.3%H_2_O) *Ammonia*	6,528.76	50	9.1·10^−9^	50	1.27		On-beam		1	[43]	
CH_4_ (N_2_+2.3%H_2_O) *Methane*	6,057.1	200	2.45·10^−8^	16	3.2		On-beam		1	[43]	
CO (N_2_+2.2%H_2_O) *Carbon monoxide*	2,176.28	100	1.48·10^−8^	71		0.002	On-beam		1	[44]	
CO (N_2_+2.6%H_2_O) *Carbon monoxide*	2,169.2	760	1.61·10^−8^	400		0.0015	On-beam		1	[45]	
N_2_O (N_2_+2.6%H_2_O) *Nitrous oxide*	2,169.6	100	2.91·10^−9^	400		0.023	On-beam		1	[45]	
CH_4_ (N_2_) *Methane*	4,245.84	770	/	1.2		1	On-beam		0.1	[46]	
CH_4_ (N_2_) *Methane*	2,958.23	770	4.06·10^−9^	1		0.1	On-beam		12	[47]	
C_2_H_2_ (N_2_) *Acetylene*	6,523.88	720	4.1·10^−9^	57		0.03	On-beam		1	[48]	
HCl (N_2_) *Hydrochloric acid*	5,739.26	760	5.2· 10^−8^	15		0.7	On-beam		1	[49]	
SO_2_ (N_2_+2.4%H_2_O) *Sulfur dioxide*	1,380.94	100	2.0·10^−8^	40		0.1		On-beam	1	[50]
C_2_H_6_ (N_2_) *Ethane*	2,990.08	150	7.0·10^−7^	217		0.025		On-beam	3	[95]
O_2_ (N_2_) *Oxygen*	13,099.3	158	4.74·10^−7^	1228		13		On-beam	1	[96]
H_2_O (air) *Water*	7,161.41	760	6.2·10^−9^	8		0.26		off-beam	1	[55]
O_3_ (air) *Ozone*	35,087.7	700	3.0·10^−8^	0.8		1.27		off-beam	1	[58]
SF_6_ *Sulfur hexafluoride*	948.62	75	2.7·10^−10^	18		5·10^−5^		fiber-coupled	1	[66]
N_2_H_4_ (N_2_) *Hydrazine*	6,570.00	700	/	62		1		MOCAM	1	[67]
C_2_H_2_ (air) *Acetylene*	6,523.88	760	1.96·10^−6^	9.8		1.58·10^4^		evanescent wave PAS	1	[75]
CH_3_OH (N_2_) *Methanol*	131.05	10	2·10^−10^	0.04		7		bare custom QTF	4	[88]
